# Polyamine and EIF5A hypusination downstream of c-Myc confers targeted therapy resistance in BRAF mutant melanoma

**DOI:** 10.1186/s12943-024-02031-w

**Published:** 2024-07-04

**Authors:** Byung-Sun Park, Heeju Jeon, Yeonseo Kim, Haejin Kwon, Ga-Eun Choi, Sung-Gil Chi, Hyun-Mee Park, Hyunbeom Lee, Tackhoon Kim

**Affiliations:** 1 Medicinal Materials Research Center, Korea Institute of Science and Technology, 5 Hwarangro-14-Gil, SeongbukGu, Seoul, 02792 Republic of Korea; 2 Department of Life Sciences, Korea University, 145 AnamRo, SeongbukGu, Seoul, 02841 Republic of Korea; 3 Center for Advanced Biomolecular Recognition, Korea Institute of Science and Technology, 5 Hwarangro- 14-Gil, SeongbukGu, Seoul, 02792 Republic of Korea; 4 Advanced Analysis and Data Center, Korea Institute of Science and Technology, 5 Hwarangro-14-Gil, SeongbukGu, Seoul, 02792 Republic of Korea; 5 Division of Bio-Medical Science and Technology, Korea University of Science and Technology, 217 GajeongRo YuseongGu, Daejeon, 34113 Republic of Korea

**Keywords:** Melanoma, BRAF, Vemurafenib, Polyamine, Hypusination, c-Myc, Drug resistance, Mitochondria

## Abstract

**Background:**

BRAF inhibitors are widely employed in the treatment of melanoma with the BRAF V600E mutation. However, the development of resistance compromises their therapeutic efficacy. Diverse genomic and transcriptomic alterations are found in BRAF inhibitor resistant melanoma, posing a pressing need for convergent, druggable target that reverse therapy resistant tumor with different resistance mechanisms.

**Methods:**

CRISPR-Cas9 screens were performed to identify novel target gene whose inhibition selectively targets A375VR, a BRAF V600E mutant cell line with acquired resistance to vemurafenib. Various in vitro and in vivo assays, including cell competition assay, water soluble tetrazolium (WST) assay, live-dead assay and xenograft assay were performed to confirm synergistic cell death. Liquid Chromatography-Mass Spectrometry analyses quantified polyamine biosynthesis and changes in proteome in vemurafenib resistant melanoma. EIF5A hypusination dependent protein translation and subsequent changes in mitochondrial biogenesis and activity were assayed by O-propargyl-puromycin labeling assay, mitotracker, mitoSOX labeling and seahorse assay. Bioinformatics analyses were used to identify the association of polyamine biosynthesis with BRAF inhibitor resistance and poor prognosis in melanoma patient cohorts.

**Results:**

We elucidate the role of polyamine biosynthesis and its regulatory mechanisms in promoting BRAF inhibitor resistance. Leveraging CRISPR-Cas9 screens, we identify AMD1 (S-adenosylmethionine decarboxylase 1), a critical enzyme for polyamine biosynthesis, as a druggable target whose inhibition reduces vemurafenib resistance. Metabolomic and proteomic analyses reveal that polyamine biosynthesis is upregulated in vemurafenib-resistant cancer, resulting in enhanced EIF5A hypusination, translation of mitochondrial proteins and oxidative phosphorylation. We also identify that sustained c-Myc levels in vemurafenib-resistant cancer are responsible for elevated polyamine biosynthesis. Inhibition of polyamine biosynthesis or c-Myc reversed vemurafenib resistance both in vitro cell line models and in vivo in a xenograft model. Polyamine biosynthesis signature is associated with poor prognosis and shorter progression free survival after BRAF/MAPK inhibitor treatment in melanoma cohorts, highlighting the clinical relevance of our findings.

**Conclusions:**

Our findings delineate the molecular mechanisms involving polyamine-EIF5A hypusination-mitochondrial respiration pathway conferring BRAF inhibitor resistance in melanoma. These targets will serve as effective therapeutic targets that can maximize the therapeutic efficacy of existing BRAF inhibitors.

**Supplementary Information:**

The online version contains supplementary material available at 10.1186/s12943-024-02031-w.

## Background

Melanoma is by far the most aggressive and lethal type of skin cancer. Activating BRAF mutations have been reported in more than 50% of melanomas, 90% of which have the V600E mutation [1]. BRAF inhibitors (BRAFi), including vemurafenib and dabrafenib, are widely used to treat patients with BRAF mutations, but resistance almost always develops. Many other pathways, including the MAPK pathway, PI3K/AKT/mTOR activation, upregulation of cyclin D1, platelet-derived growth factor beta (PDGFR) and epidermal growth factor receptor (EGFR), were found to be associated with BRAFi resistance [2]. MEK inhibitors (MAPKi), such as cobimetinib or trametinib, and immune checkpoint inhibitors such as atezolizumab are used in clinic in combination with BRAF inhibitors to delay the onset of drug-resistant tumor outgrowth [3, 4]. However, recent findings showed that BRAFi resistant melanoma responds more poorly to MEK and immune checkpoint inhibitors [5, 6]. Effective, druggable approaches that target vulnerabilities common to broad range of the diverse and heterogeneous nature of the mechanisms underlying BRAFi resistance will help combat therapy-resistant cancer.

Polyamines, which encompass putrescine, spermidine and spermine, are abundant low-molecular-weight metabolites known to regulate cell proliferation, cell differentiation, and DNA stability [7]. These classic oncometabolites have long been recognized to be enriched in the urine and plasma of cancer patients [8]. Polyamines contribute to cancer progression by interacting with the mTOR [9], RAS [10], and AKT pathways [11]. Particularly, spermidine acts as an aminobutyl group donor for the hypusination of the EIF5A protein by deoxyhypusine synthase (DHPS) and deoxyhypusine hydroxylase (DOHH) [12]. Hypusinated EIF5A facilitates the translational elongation of specific mRNA transcripts encoding mitochondrial proteins [13] or proteins containing polyproline [14]. Although polyamine modulation has been proposed as a promising cancer therapy [15], clinical trials with polyamine biosynthesis inhibitors, including the ornithine decarboxylase inhibitor eflornithine (DL-α-difluoromethylornithine, DFMO), have shown only modest antineoplastic activity. For example, only one out of 21 patients with metastatic melanoma experienced a complete response by DFMO treatment in phase II clinical trial [16]. It has been proposed that polyamines in diet and those synthesized from commensal bacteria significantly undermine the therapeutic efficacy of polyamine biosynthesis inhibitors. To this end, combining a polyamine uptake blocker with a polyamine biosynthesis inhibitor has proven effective against BRAF mutated melanoma [17]. For example, polyamine transport inhibitor AMXT-1501 [18], in combination with DFMO, is undergoing clinical trials against solid cancer (NCT05500508). In addition, inhibiting the major downstream effector function of polyamines, mainly EIF5A hypusination, has recently gathered considerable interest as an effective cancer therapy [19]. Understanding the molecular mechanisms underlying the contribution of polyamines and EIF5A hypusination to tumorigenesis will help identifying tumors most effectively targetable with polyamine biosynthesis and EIF5A hypusination inhibitors for maximal therapeutic benefit.

Recent studies have revealed extensive metabolic rewiring, including increase in mitochondrial biogenesis and oxidative phosphorylation (OXPHOS) activity [20–22] in BRAFi and MAPKi treated melanoma. BRAF inhibitors activate the transcription of microphthalmia-associated transcription factor (MITF), which, in turn, promotes the expression of peroxisome proliferator-activated receptor gamma coactivator 1-alpha (PGC1α), a master regulator of mitochondrial biogenesis [23]. PGC1α promotes drug resistance against MAPK inhibitors [21] or ROS-inducing drugs [24]. Other mechanisms, including JARID1B-mediated epigenetic regulation, have been associated with the upregulation of mitochondrial OXPHOS [25] in slow-cycling, intrinsically drug-resistant melanoma. BRAFi and MAPKi treatment induces a transcriptional state mimicking nutrient starvation, leading to a reduction in glucose uptake and glycolysis. This drives activation of mitochondrial fatty acid oxidase (FAO) to maintain cellular viability [26, 27]. Glutamine dependence is also observed in BRAFi resistant cell populations [28]. These evidences suggest an intriguing possibility that targeting cellular metabolism may be an effective therapeutic strategies preventing emergence of therapy resistant melanoma.

In this study, through CRISPR-Cas9 screening, we identified AMD1 as a critical enzyme for acquiring vemurafenib resistance by upregulating polyamine biosynthesis. Increased polyamine biosynthesis and EIF5A hypusination contribute to vemurafenib resistance in A375 melanoma by enhancing mitochondrial activity. We further demonstrate that persistent activation of c-Myc is responsible for the activation of polyamine synthesis in vemurafenib-resistant cancer. Inhibition of polyamine biosynthesis, EIF5A hypusination, or c-Myc suppressed vemurafenib resistance in melanoma cell line models with either acquired or intrinsic resistance against BRAF inhibitors both in vitro and in vivo. Collectively, our results highlight the polyamine biosynthesis-EIF5A hypusination-mitochondrial activity axis modulated by c-Myc as a promising target to overcome vemurafenib resistance in melanoma.

## Methods

### Cell Culture

HEK293T, SK-mel-28, and Hs294T cells were cultured in Dulbecco’s modified Eagle’s medium (DMEM) supplemented with 10% fetal bovine serum (FBS) and antibiotics. A375 cells were cultured in RPMI 1640 with 10% FBS and antibiotics. All cell lines were purchased from Korean Cell Line Bank (https://cellbank.snu.ac.kr). A375VR and SK-mel-28VR cells with acquired vemurafenib resistance were generated by initial treatment of 50nM vemurafenib and escalating vemurafenib dose by two-fold each week. The terminal vemurafenib dose was 2µM and 200nM for A375VR and SK-mel-28VR, respectively.

### CRISPR screens & next-generation sequencing (NGS) analysis

Library construction was prepared as previously described [29]. The druggable gene library contained 6935 sgRNAs targeting 2305 genes and hU6-sgRNA cassette was cloned into FUW-EFS-PuroR lentiviral vector. The lentiviral library vector was co-transfected with psPAX2 and pVSV-G vectors with Polyethyleneimine (Polysciences Inc.) for lentivirus production. A375-Cas9 and A375VR-Cas9 cell lines were transduced at a multiplicity of infection (MOI) at 0.4–0.5. All cells were passaged with fold coverage of at least 1000 in the presence of puromycin (2 µg/µl). After incubation for 3 weeks, all cells were harvested for genomic DNA isolation using Accuprep Genomic DNA Isolation Kit (Bioneer). NGS was performed by HiSeq2500 with a 100 bp-paired end.

### Gene knockout using CRISPR‒Cas9

sgRNAs targeting genes (AMD1, ODC1, DHPS, PGC1α) for knockout were cloned and inserted into U6 promoter-containing lentiviral vectors (FUW-EFS-PuroR) for virus production. After 2 days of transduction into Cas9-expressing cells, puromycin (2 µg/µl) was added for 5–14 days for selection. The list of sgRNAs used in the study are listed in Table S1.

### GFP competition assay

The GFP competition assay was performed as previously described [29]. Briefly, gRNA was cloned and inserted into the FUW-EFS-GFP vector. Five days after viral transduction into Cas9-expressing cells, the fraction of GFP-positive cells was quantified using BD Accuri C6 on Day 0, Day 7, and Day 14.

### Quantification of mRNA expression levels

TRIzol™ Reagent (Invitrogen) was used for RNA isolation according to the manufacturer’s instructions. cDNA synthesized using M-MLV reverse transcriptase (Enzynomics) with random hexamers was subjected to real-time PCR using a Step-One real-time PCR system (Applied Biosystems). The primers used for the assay are listed in Table S2.

### Western blot

All samples were lysed with RIPA buffer (50 mM Tris-Cl pH 7.5, 1% Nonidet P-40, 0.5% sodium deoxycholate, 0.1% sodium dodecyl sulfate, 150 mM sodium chloride, 1 mM EDTA) containing protease and phosphatase inhibitors. The protein concentrations were determined using a BCA assay (Thermo Fisher Scientific) for SDS‒PAGE analysis. The primary antibodies used in this study were as follows: AMD1 (Proteintech, 11052-1-AP), c-Myc (Cell Signaling, D84C12), EIF5A (Santa Cruz, sc-390,202), EIF5A^H^ (Creative Biolabs, clone Hpu24), ACTB (Santa Cruz, sc-8432), NDUFA9 (Invitrogen, 20C11B11B11) and MDH2 (Cell Signaling, D8Q5S).

### Cell viability assay for drug treatment and evaluation of drug synergy

All cells were seeded at 1000–2000 cells/well in 96-well plates, treated with drug on the next day and further incubated for 72 h. Cell viability was measured by incubating with EZ-Cytox solution (DogenBio) diluted in fresh medium at the end of drug treatment at 37 °C for 2–4 h. The absorbance at 450 nm was measured with a Wallac EnVision Multilabel Plate Reader (Perkin Elmer). Drug synergy was evaluated with the Synergyfinder [30] web application (https://synergyfinder.fimm.fi/).

### Live/dead staining assay

A live/dead staining assay was performed using a live/dead viability/cytotoxicity kit (Invitrogen) according to the manufacturer’s instructions. Briefly, A375VR cells were seeded in 24-well plates (20,000 cells/well). Drugs were treated on the next day for trypsinization and harvesting 2 days later. Cells were treated with calcein AM and ethidium homodimer-1 (live/dead viability/cytotoxicity kit [Invitrogen]) according to the manufacturer’s instructions and were analyzed by BD Accuri C6.

### Xenograft

All animal experiments were approved by IACUC of Korea Institute of Science and Technology (KIST). Five-week-old BALB/c nude mice (Dooyeol Biotech) were subcutaneously injected with A375VR cells (1 × 10^7^ cells). Vemurafenib (20 mg/kg) dissolved in 45% PEG300 + 10% DMSO + 45% saline was intraperitoneally injected daily starting from the day the average tumor volume reached 100mm^3^ until the end of experiment. DFMO (Biosynth) was supplied in drinking water (2% w/v). The tumor volumes were measured for two weeks since the start of drug administration with digital calipers.

### Mass spectrometry for quantification of polyamines

Cell pellets were treated with 1 mL of 70 mM HEPES in 60% cold MeOH, including 2 µM d_5_-glutamine as an internal standard. Cells were lysed by running 3 freeze/thaw cycles using liquid nitrogen. Then, the lysate was centrifuged at 14,000 rpm for 10 min at 4 °C. The supernatant was transferred into new centrifuge tubes and used for metabolite analysis or otherwise stored at -80 °C before use. For normalization, 5 µL of supernatant from each sample was used to quantitate DNA concentration using a Nano-MD UV‒Vis spectrophotometer (Scinco, Seoul, Korea). For metabolite analysis, 400 µL of the supernatant from each sample was evaporated to dryness at 37 °C under nitrogen. Phenylisothiocyanate (PITC) derivatization was performed by adding 50 µL of a mixture of 19:19:19:3 ethanol: water: pyridine: PITC (v/v). The mixture was gently vortexed for 30 s, shaken for 20 min at room temperature, and then evaporated under nitrogen for 1 h at 37 °C. Then, the residue was reconstituted by adding 100 µl of mobile phase A: B = 5:5 and vortexed for 1 min. The reconstituted samples were injected into the LC‒MS/MS for analysis.

An LC‒MS/MS consisting of an Exion LC Series UPLC (AB Sciex, Framingham, USA) and a 4500 Triple Quad mass spectrometer (AB Sciex, Framingham, USA) equipped with an electrospray ionization source (ESI) was used. Column and autosampler temperatures were maintained at 50 °C and 4 °C, respectively, and the injection volume was 5 µL. The analytes were separated by an Acquity UPLC BEH C18 column (1.7 μm, 2.1 mm x 75 mm, Waters, USA). The mobile phase was 0.2% formic acid in deionized water (A) and 0.2% formic acid in acetonitrile (B). During the analysis, the flow rate was maintained at 0.4 mL/min and was run as a gradient elution. The initial mobile phase conditions were 100% solvent A. After 0.9 min, solvent B reached 15% over 4.1 min. Solvent B then reached 70% in 5 min and was set to 100% over 0.5 min. Then, 100% solvent B was maintained for 2.3 min and returned to the initial conditions for 0.2 min. It was re-equilibrated for 2 min at the initial conditions. The total running time for each sample was 15 min.

Mass spectrometry was performed using the positive ionization mode. The ionization conditions of the mass spectrometer were as follows. Ion spray voltage, 5.5 kV; source temperature, 500 °C; curtain gas, 45 psi; collision gas, 9 psi; nebulizer gas, 60 psi; turbo gas, 70 psi. All analytes were detected in multiple reaction monitoring (MRM) mode, and analysis data collection and processing were performed using Analyst 1.6.2 software (AB Sciex, Framingham, USA).

### Mass spectrometry for proteomic analysis

All samples were lysed with RIPA lysis and extraction buffer (Thermo Scientific, 89,900), and the solvent was changed to 50 mM ammonium bicarbonate buffer using a 10 K MWCO filter (Amicon, UFC5010). Quantification of proteins in the sample was performed using a Qubit Protein Assay Kit (Thermo Scientific, Q33212). Following that, samples (100 µg) were reduced and alkylated via treatment with reduction buffer (100 mM dithiothreitol) at 60 ℃ for 45 min and alkylation buffer (200 mM iodoacetamide) at room temperature for 45 min. Then, samples were digested by using trypsin (10 µg) at 37 ℃ overnight and dried using a SpeedVac concentrator (Labogene, HyperVAC). Finally, the salts in the sample were removed using a C18 microspin column (Harvard Apparatus, 74-4601).

Tryptic peptides were analyzed with an Orbitrap Tribrid mass spectrometer (Eclipse model, Thermo Fisher Scientific, San Jose, USA) coupled with an Ultimate 3000 nano-LC system (Thermo Fisher Scientific, USA). Peptides were dissolved in 100 µL of buffer A (0.1% formic acid in distilled water (DW)), and 5 µL was injected into the nanoelectrospray ion source. Injected samples were loaded onto a trap column (Acclaim PepMap C18 nano Viper 100, 75 μm x 2 cm, 3 μm, Thermo Fisher Scientific) at a flow rate of 5 µL/min with 95% buffer A. After 4 min, peptides were separated on an analytical column (PepMap RSLC C18 ES803A, 2 μm, 75 μm x 50 cm, USA) by a 150 min gradient from 5 to 90% solvent B (0.1% formic acid in acetonitrile) at a flow rate of 300 nL/min. Data quality was evaluated with a HeLa protein digest standard (100 ng, cat # 88,328, Thermo Fisher Scientific) throughout the sequence. The Tribrid Orbitrap mass spectrometer was operated in a data-dependent Top20 scan mode switching between MS and MS2. The following parameters were used for MS acquisition: mass accuracy, 10 ppm; ion spray voltage, 1850 V; capillary temperature, 275 °C; and resolution of full scans (m/z 375–1575), 120,000. HCD activation scans were acquired with 35% normalized collision energy (NCE). The quadrupole isolation window was 1.4 Da. MS/MS spectra were detected on Orbitrap with a resolution of 30,000.

Raw data from MS were processed from MaxQuant (Quantitative proteomics software, Max Planck Institute of Biochemistry) using UniProt Human DB (11 May 2022 ver.) and annotated by the MaxLFQ algorithm for label-free quantification (LFQ). Perseus 2.0.6 was used as the statistical tool for normalization, transformation and p value calculation.

### OPP-labeled protein pull-down assay

OPP-labeled nascent proteins were detected by immunoblot analysis as described previously [31]. A375 parental or A375VR cells treated with drugs were incubated with fresh medium containing 30 µM OPP for 3 h. Cells were then washed with cold PBS and lysed with RIPA buffer containing protease and phosphatase inhibitors. Then, OPP-tagged proteins were conjugated with biotinylated azide (Click Chemistry Tools) by using a click reaction with a Click-&-Go® Protein Reaction Buffer Kit according to the manufacturer’s instructions. The biotin-conjugated protein samples were precipitated overnight at -20 °C by adding 5 volumes of cold acetone. The precipitated proteins were pelleted and washed twice with cold methanol. Five hundred micrograms of precipitated proteins resuspended in RIPA with 1% SDS were incubated with streptavidin magnetic beads overnight at 4 °C with rotation. The next day, the samples were washed 3 times with cold NETN buffer (20 mM Tris-Cl pH 8.0, 0.5% Nonidet P-40, 100 mM sodium chloride, 1 mM EDTA), and streptavidin bead binding proteins were analyzed with SDS‒PAGE.

### Reporter assay for quantification of translation rate

A375 and A375VR cells were transduced with retrovirus generated with the pMSCV Puro plasmid (Clontech) containing the reporter gene described in Fig. 3F. GFP and miRFP signals were detected by BD Accuri C6. The miRFPnano3 gene [32] (depicted as miRFP in Fig. 3F) was used as an internal control for normalization.

### MitoTracker & MitoSOX assay

Cells were trypsinized and counted to stain equal numbers of cells with MitoTracker (500 nM, Cell Signaling Technology) diluted in growth medium or mitoSOX red (1 µM, MedChemExpress) diluted in PBS at room temperature for 15 min in the dark. Cells were washed and resuspended in PBS for analysis with BD Accuri C6 and its accompanying software.

### Seahorse assay

A375 or A375VR cells were seeded in XFe8 plates at 6000 cells per well in minimally buffered XF RPMI medium supplemented with 10 mM glucose and 2 mM pyruvate. The next day, DMSO or vemurafenib (1 µM) was added for 24 h, and the OCR was measured by a Seahorse XF analyzer (Agilent) by sequential addition of oligomycin (0.5 µM), carbonyl cyanide 4-(trifluoromethoxy) phenylhydrazone (FCCP) (1 µM), and rotenone/antimycin A (0.5 µM) as recommended in the manufacturer’s instructions.

### Public database analysis

Gene Expression Omnibus (GEO) data with BRAFi or BRAFi + MAPKi treated melanoma patient cohort (GSE65185 [5], GSE61992 [33]) were analyzed after quantile normalization using preprocessCore (v1.66.0) package.

### Statistics

Statistical analyses were performed using Graphpad Prism 9 (GraphPad, USA). Student’s t test was used for two-group comparisons unless otherwise stated. p value of < 0.05 was marked as statistically significant.

## Results

### CRISPR-Cas9 screening identifies AMD1 as a sensitizing factor for vemurafenib resistance

**Fig. 1:**
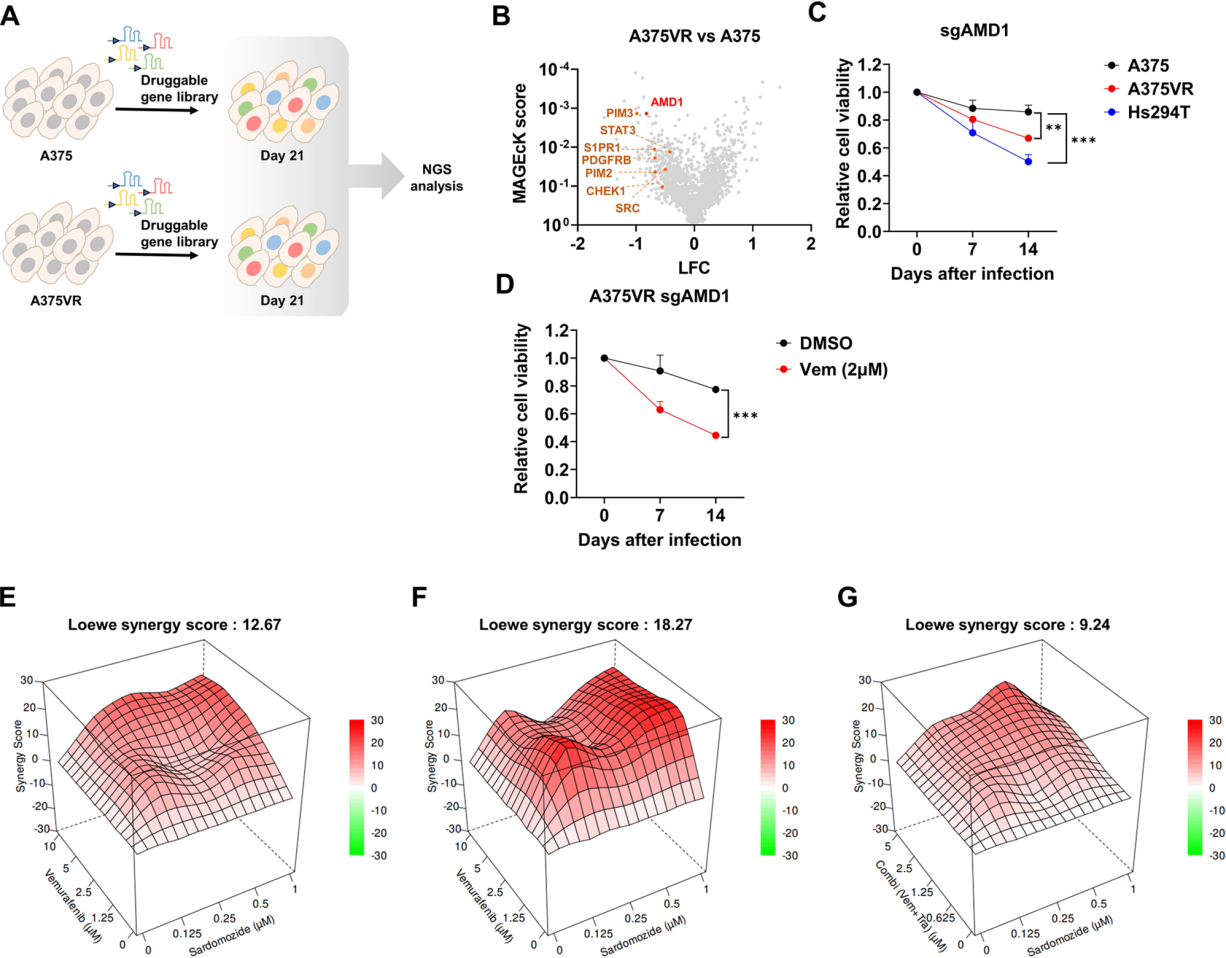
AMD1 inactivation sensitizes BRAF mutant melanoma to vemurafenib. **A** Schematic diagram of CRISPR-Cas9 screening used in this study. **B** Volcano plot analyzed by MAGeCK. **C** GFP competition assay using GFP-sgAMD1 expression construct in indicated cell lines (*n* = 3). **D** GFP competition assay in A375VR treated with DMSO or vemurafenib (*n* = 3). **E-F** Drug synergy score of vemurafenib and sardomozide calculated by SynergyFinder using Loewe model in **E** A375VR, and **F** Hs294T. **G** Drug synergy score of vemurafenib + trametinib combination and sardomozide in A375VR. Vemurafenib and Trametinib were treated as 2-fold dilution starting from 5µM and 5nM, respectively. All drugs were treated for 72 h (**E-G**). All plots indicate mean ± s.d. Student’s t-test was used to determine statistical significance for **C** and **D**. *, *p* < 0.05; **, *p* < 0.01; ***,*p* < 0.001; ****,*p* < 0.0001

We performed a CRISPR-Cas9 loss-of-function screening to identify genes whose ablation can attenuate vemurafenib resistance in the BRAF V600E mutant A375 melanoma model. The A375 vemurafenib-resistant (A375VR) cell line was generated by treating A375 parental cells with an escalating dose of vemurafenib for 2 months and pooling the outgrown cells (Fig. S1A, see methods). A375VR cells showed modest increases in expression of BRAF and receptor tyrosine kinases such as IGF1R and MET, G13R point mutation in NRAS gene and Notch signaling activation (Figs. S1B-C). There were no detectable BRAF splice variants yielding N-terminal truncated BRAF protein [34] (Fig. S1D). Consistent with previous studies [35], both AKT and ERK activities were sustained even after vemurafenib treatment in A375VR (Fig. S1E). AKT inhibitor MK2206, but not ERK inhibitor SCH722984, significantly attenuated resistance in A375VR model (Fig. S1F), suggesting that our A375VR model is at least partly dependent on sustained AKT activity for vemurafenib resistance. We delivered a sgRNA library targeting a set of druggable genes [36] into A375 parental cells and A375VR cells stably expressing Cas9 to identify sgRNAs specifically depleted in A375VR using next-generation sequencing (Fig. 1A). The quality of the sgRNA library and reproducibility of the sgRNA frequency between replicates were validated (Figs. S2A-B). MAGeCK analysis [37] revealed significant depletion of sgRNAs targeting known resistance driver genes, including PIM kinase [38], S1PR1 [39], PDGFRB [40], CHEK1 [41], SRC [42] and STAT3 [43]^,^ supporting the robustness of our screens (Fig. 1B). AMD1 (S-adenosylmethionine decarboxylase) was identified as one of the top hits depleted in A375VR cells in our screen. Consistently, a cell competition assay using flow cytometry [29] confirmed that AMD1 knockout selectively decreased the viability of A375VR and Hs294T (a BRAF V600E mutant cell line with intrinsic resistance to vemurafenib), while vemurafenib-sensitive parental A375 cells were less affected (Fig. 1C). Genetic ablation of AMD1 by CRISPR-Cas9 (Fig. 1D, S3A) or pharmacological inhibition of AMD1 with sardomozide [44] (Fig. 1E) sensitized A375VR cells to vemurafenib. We similarly confirmed AMD1 dependence in independent cell lines Hs294T (intrinsically vemurafenib-resistant; Fig. 1F) and with SK-mel-28VR (acquired vemurafenib-resistant; Fig. S3B). SK-mel-28VR cell line was generated analogously to A375VR from SK-mel-28, and has partly AKT dependent acquired vemurafenib resistance (Figs. S1B-F). Vemurafenib is often used in combination with MEK inhibitors. We therefore asked whether AMD1 inhibition can also sensitize melanoma to a combination of vemurafenib and MEK inhibitor trametinib. Notably, consistent with previous observations [2], our cell lines with acquired vemurafenib resistance already developed resistance to trametinib (Figs. S3C-D). As expected, pharmacological inhibition of AMD1 sensitized A375VR to the vemurafenib/trametinib combination (Fig. 1G).

### Elevated polyamine biosynthesis and EIF5A hypusination are necessary and sufficient for vemurafenib resistance

**Fig. 2:**
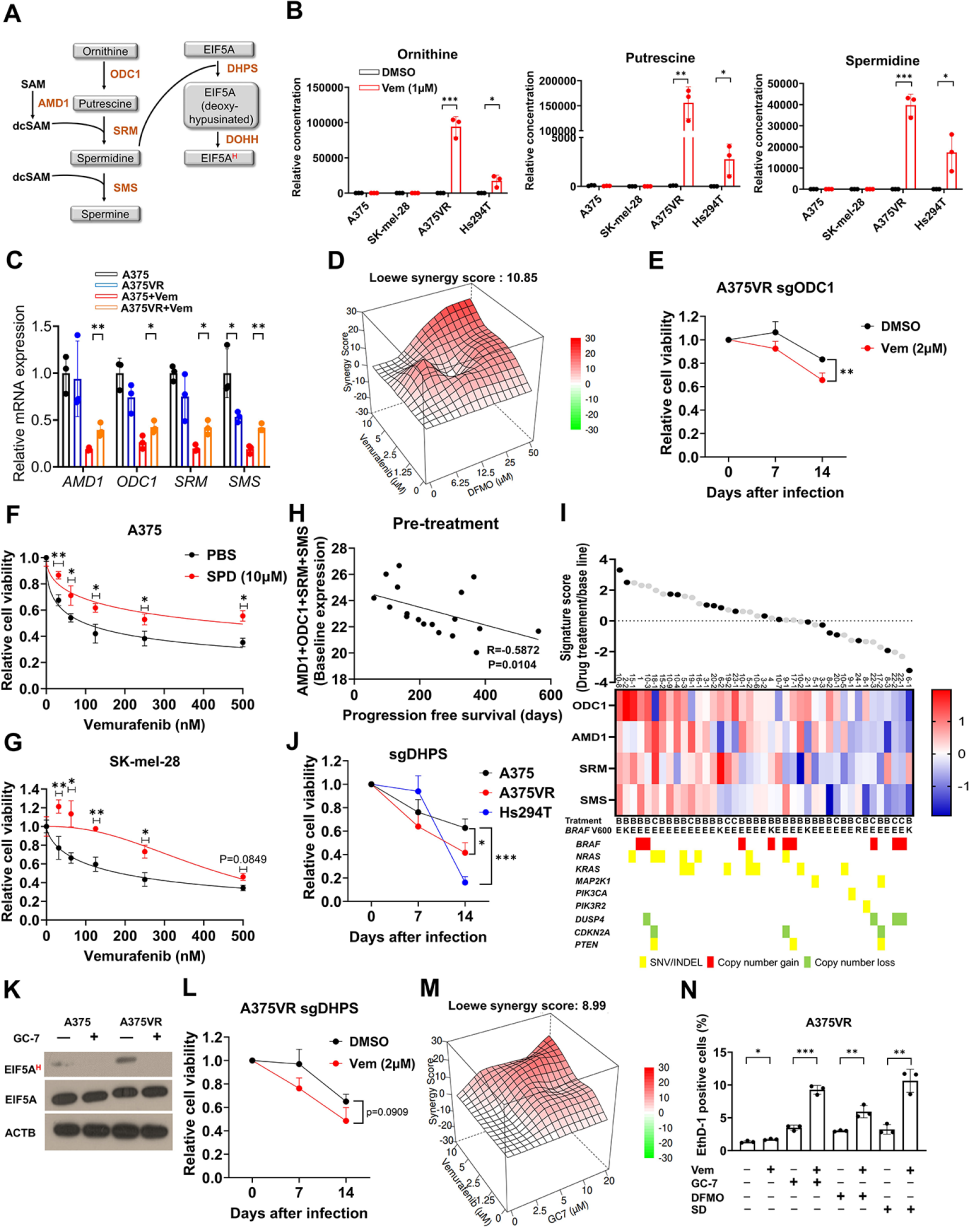
Polyamine synthesis is upregulated and critical in vemurafenib resistance. **A** Schematic diagram of polyamine biosynthesis pathway. **B** quantification of ornithine, putrescine and spermidine in melanoma cell lines (*n* = 3). All cells were treated with vemurafenib (1µM) or DMSO for 48 h and harvested for polyamine quantification (see methods). **C** Relative mRNA expressions of polyamine synthesis genes in A375 and A375VR after 24 h of vemurafenib treatment (*n* = 3). **D** Drug synergy score (using Loewe model) of vemurafenib and DFMO in A375VR. **E** GFP competition assay using GFP-sgODC1 in A375VR treated with vemurafenib (*n* = 3). **F-G** Vemurafenib dose response curve of indicated cells with or without spermidine supplementation (*n* = 3). Vemurafenib and spermidine were treated for 72 h. **H** Correlation between progression free survival and the expression of polyamine synthesis genes before drug treatment in melanoma patient cohort described in Hugo et. al [5]. Gene expression is presented as log fragments per kilobase of transcript per million (FPKM). Correlation coefficient is calculated as non-parametric Spearman’s r. **I** Log fold changes (LFC) of polyamine synthesis related genes after indicated drug treatment in patient cohort described in (H). Black circles indicate patient samples of harboring no gene mutations causing MAPKi resistance). **J** GFP competition assay using GFP-sgDHPS in indicated cell lines (*n* = 3). **K** Immunoblots of EIF5A hypusination in A375 and A375VR treated with GC-7 for 48 h. **L** GFP competition assay using GFP-sgDHPS in A375VR treated with vemurafenib (*n* = 3). **M** Drug synergy score (using Loewe model) of vemurafenib and GC-7 in A375VR. **N** Quantification of cell death with A375VR cells treated with indicated drug combinations for 24 h using Live/dead cell staining assay. Vemurafenib: 2µM, GC-7: 5µM, DFMO: 100µM, and SD (sardomozide): 0.5µM (*n* = 3). All plots indicate mean ± s.d. Student’s t-test was used to determine statistical significance for **B-C**, **E-G, J, L** and **N**. *, *p* < 0.05; **, *p* < 0.01; ***,*p* < 0.001; ****,*p* < 0.0001

AMD1 is an essential enzyme in the biosynthesis of polyamines (Fig. 2A). Therefore, we asked whether vemurafenib resistance is associated with increased polyamine biosynthesis. Intriguingly, vemurafenib treatment dramatically upregulated ornithine, putrescine and spermidine in A375VR cells but not in the A375 parental cell line (Fig. 2B). This increase may be attributed to sustained MAPK and AKT signaling in vemurafenib resistant cells (see discussion). While the mRNA expression levels of polyamine biosynthesis enzymes were comparable between parental and vemurafenib resistant A375 cells at baseline, their levels were sustained at higher level in A375VR cells compared with parental A375 cells when treated with vemurafenib (Fig. 2C). This led us to test whether polyamines directly contribute to vemurafenib resistance. Treatment of DFMO, a specific inhibitor of ornithine decarboxylase (ODC1), a rate-limiting enzyme in putrescine biosynthesis, or genetic ablation of ODC1 by CRISPR-Cas9 sensitized A375VR, SK-mel-28VR and Hs294T cells to vemurafenib or a combination of vemurafenib and trametinib (Fig. 2D-E, S3E-G). Conversely, spermidine supplementation induced vemurafenib resistance in parental vemurafenib-sensitive A375 and SK-mel-28 cells (Fig. 2F-G).

We next examined the clinical relevance of our finding by analyzing RNA sequencing data in a BRAF mutant melanoma patient cohort treated with BRAF and/or MEK inhibitors (BRAFi and MAPKi) [5]. Interestingly, higher expression of *AMD1, ODC1, SRM* and *SMS* was correlated with shorter progression-free survival after BRAF/MAPK inhibitor treatment (Fig. 2H). Additionally, recurrent, therapy-resistant tumors after BRAF/MAPK inhibitor treatment had higher polyamine biosynthesis signature scores (consisting of expression levels of *AMD1, ODC1, SRM*, and *SMS*) than treatment-naïve tumors (Fig. 2I) in 61% (25/41) of cases. Notably, the polyamine biosynthesis signature in patient samples after BRAFi or BRAFi/MAPKi treatment was also enriched in tumors without common genomic alterations such as BRAF amplification and oncogenic RAS missense mutations. Our findings suggest polyamine biosynthesis as a vemurafenib resistance mechanism that cannot be solely attributed to established genetic alterations that confer treatment resistance [45]. Similar enrichment of polyamine biosynthesis signature in 73% (8/11) of therapy-resistant tumors was observed in an independent cohort treated with dabrafenib and trametinib [33] (Fig. S3H).

One of the major roles of polyamine is EIF5A hypusination, a unique posttranslational modification involving covalent conjugation of the aminobutyl group of spermidine [12]. Hypusinated EIF5A, as a part of eukaryotic translation initiation factors, enhances the translation of mRNAs that are inefficiently translated with unmodified EIF5A. Intriguingly, ablation of deoxyhypusine synthase (DHPS), the rate-limiting enzyme in EIF5A hypusination (Fig. 2A), selectively reduced cell viability in vemurafenib-resistant A375VR and Hs294T cells while sparing vemurafenib-sensitive A375 parental cells (Fig. 2J). Indeed, EIF5A hypusination was increased in A375VR cells compared to its parental cells (Fig. 2K). Inhibition of DHPS by treatment of specific inhibitor GC-7, completely abrogated EIF5A hypusination, confirming that increased EIF5A hypusination in A735VR is completely dependent on DHPS. DHPS knockout or treatment with GC-7, sensitized A375VR, Hs294T and SK-mel-28VR cells to vemurafenib or vemurafenib + trametinib (Fig. 2L-M, S3G, S3I-J). The synergistic cell death by treatment with either GC-7, DFMO, or sardomozide in combination with vemurafenib was also confirmed with a live/dead assay (Fig. 2N).

A recent study revealed that targeting fatty acid oxidation by ranolazine delays tumor recurrence with acquired BRAFi resistance by rewiring methionine salvage pathway, leading to upregulation of polyamine biosynthesis [27]. Treatment of ranolazine increased the expression of interferons and genes involved in antigen presentations, such as *B2M* and *TAP1*. This raises concerns that the increase in polyamine increases tumor immunogenicity, and conversely, inhibition of polyamine biosynthesis may render tumor cells less immunogenic, thereby making tumor more refractory to immune checkpoint blockade. However, treatment of A375VR cells with GC-7 or DFMO increased antigen presentation genes and interferons (Fig. S3K), suggesting that inhibition of polyamine biosynthesis or EIF5A hypusination may enhance the immunogenicity of tumors. The discrepancy between our findings and the previous study may be explained by the fact that upregulation of polyamine is one of the many consequences by metabolic rewiring induced by ranolazine treatment, which may include fatty acid oxidation and glutathione synthesis and nucleotide biosynthesis downstream of methionine salvage pathway, so the increase in interferon and antigen presentation genes may not entirely result from the increase in polyamines. Indeed, previous studies revealed that polyamine biosynthesis and uptake inhibition sensitized 4T1 syngeneic tumor model to immune checkpoint blockade [15, 46], suggesting that, in line with our data, inhibition of polyamine may also attenuate resistance against immune checkpoint inhibitor.

#### EIF5A hypusination-dependent translation upregulation of mitochondrial proteins induces vemurafenib resistance

**Fig. 3:**
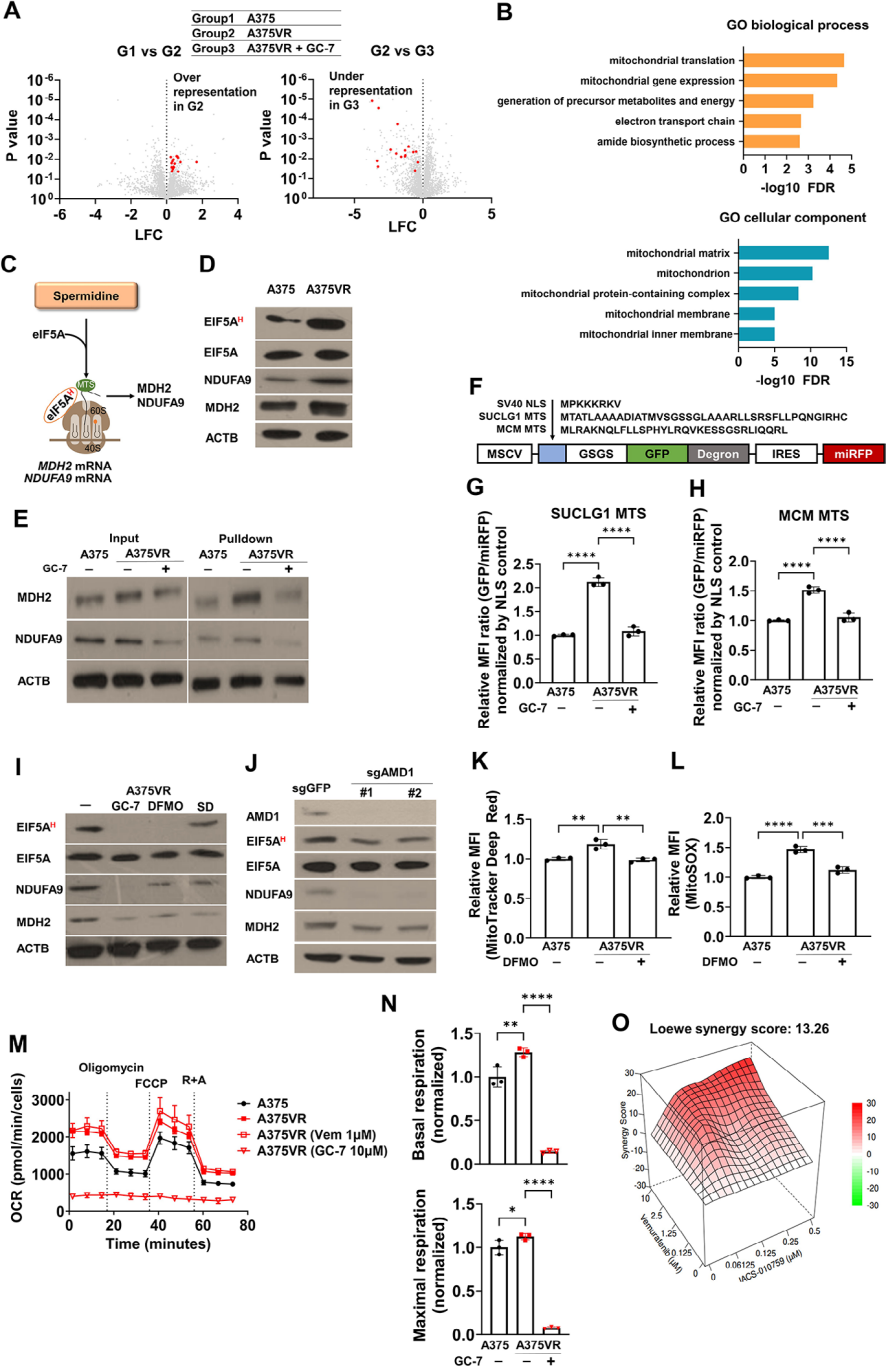
Hypusination and upregulation of mitochondrial respiration are critical for vemurafenib resistance. **A** Differential proteomic analysis between indicated groups using mass spectrometry. Mitochondrial proteins are highlighted in red. **B** Gene ontology analysis of proteins significantly upregulated in group 2 compared to both groups 1 and 3 in (A). **C** Schematic diagram of protein translation accelerated by hypusinated EIF5A. **D** Immunoblots of EIF5A hypusination and 2 mitochondrial proteins. **E** O-propargyl-puromycin (OPP)-labeled pull down assay of indicated proteins. GC-7 (10µM) was treated for 48 h for cell harvest, and PBS was treated for negative control of GC-7. **F** Schematic diagram of reporter gene for analyzing translation rate of protein containing mitochondrial targeting sequence (MTS). **G-H** Reporter assay described in (F) with indicated MTS in the presence or absence of GC-7 (10µM) in A375VR (*n* = 3). **I** Western blot analysis of EIF5A hypusination and mitochondrial proteins in A375VR cells treated with indicated drugs for 48 h. GC-7: 10µM, DFMO: 200 µM, SD (Sardomozide): 2µM. **J** western blot analysis of EIF5A hypusination and mitochondrial proteins in A375VR-Cas9 cells expressing indicated sgRNAs. **K-L** Mitotracker deep red staining (K) and MitoSOX staining (L) with A375 and A375VR cells treated with DFMO (100µM) or PBS (*n* = 3). **M** Oxygen consumption rate of A375 and A375VR cells treated with indicated drug for 48 h (*n* = 3). **N** Basal respiration and maximal respiration data from (M). **O** Drug synergy score (using Loewe model) of vemurafenib and IACS-010759 combination in A375VR. Vemurafenib and IACS-010759 were treated for 72 h. One way ANOVA was used for testing statistical significance unless otherwise indicated for **G-H, K-L** and **N**. All plots indicate mean ± s.d. *, *p* < 0.05; **, *p* < 0.01; ***,*p* < 0.001; ****,*p* < 0.0001

EIF5A hypusination enhances the translation of specific proteins including proteins with a stretch of polyproline [14] or mitochondrial proteins [13]. Therefore, the consequence of EIF5A hypusination in protein translation was examined by proteomic analysis using mass spectrometry with A375 and A375VR. Strikingly, multiple mitochondrial proteins, including MRPL37, MRPL11, MDH2 and NDUFA9, were strongly enriched in A375VR cells compared to their parental cells, and this difference was abrogated upon DHPS inhibition by GC-7 (Fig. 3A). Gene ontology analysis also confirmed the overrepresentation of mitochondrial proteins in A375VR cells (Fig. 3B, Table S3). In line with this, the mitochondrial proteins NDUFA9 and MDH2 were increased in A375VR without any significant changes in their mRNA levels (Fig. 3C-D, S4A). The increase in EIF5A hypusination NDUFA9 and MDH2 were similarly confirmed in SK-mel-28VR and Hs294T (Fig. S4B-C). Analysis of nascent proteins by O-propargyl-puromycin (OPP) labeling assay [31] confirmed the upregulation of NDUFA9 and MDH2 protein translation in A375VR (Fig. 3E). Expectedly, GC-7 treatment significantly decreased NDUFA9 and MDH2 nascent protein levels, suggesting that enhanced mitochondrial protein translation was dependent on DHPS activity and EIF5A hypusination. Previous study revealed that EIF5A hypusination increases mitochondrial protein translation by enhancing translation efficiency of RNA encoding mitochondrial targeting sequence (MTS) [13]. To this end, we constructed a fluorescent reporter tagged with MTS to monitor EIF5A hypusination-dependent translation of MTS-tagged proteins (Fig. 3F). As a result, GFP reporters tagged with two independent MTSs were selectively upregulated in A375VR and this increase was reversed by GC-7 treatment (Fig. 3G-H).

EIF5A hypusination dependency of mitochondrial protein translation suggested that decreased mitochondrial biogenesis and function may be responsible for polyamine mediated BRAFi resistance in melanoma. Indeed, treatment of A375VR and SK-mel-28VR cells with GC-7, DFMO or sardomozide decreased EIF5A hypusination and mitochondrial protein expression (Fig. 3I, S4D). Additionally, AMD1 knockout decreased EIF5A hypusination and mitochondrial protein expression (Fig. 3J). Conversely, spermidine supplementation of A375 parental cells increased EIF5A hypusination in mitochondrial proteins (Figs. S4E-F). In line with this, spermidine supplementation attenuated the synergistic effects of vemurafenib and polyamine synthesis inhibitors (Fig. S4G). In addition, wild-type EIF5A overexpression promoted vemurafenib resistance in A375 cells, while non-hypusinatable EIF5A K50A or K50R mutant overexpression failed to do so [47] (Figs. S4H-I). Mitotracker and mitoSOX staining revealed that mitochondrial content and mitochondrial superoxide were increased in A375VR cells, and this increase was abrogated by DFMO treatment (Fig. 3K-L). The mitochondrial oxygen consumption rate was also increased in A375VR cells (Fig. 3M-N). Consistently, treatment with IACS-010759 [48], an inhibitor of OXPHOS, synergized with vemurafenib in A375VR, Hs294T and SK-mel-28VR cells (Fig. 3O, S4J). Similar synergistic cell death was observed with triple combination of IACS-010759 and vemurafenib and trametinib (Fig. S4J).

### c-Myc reprograms A375VR cells to gain vemurafenib resistance

**Fig. 4:**
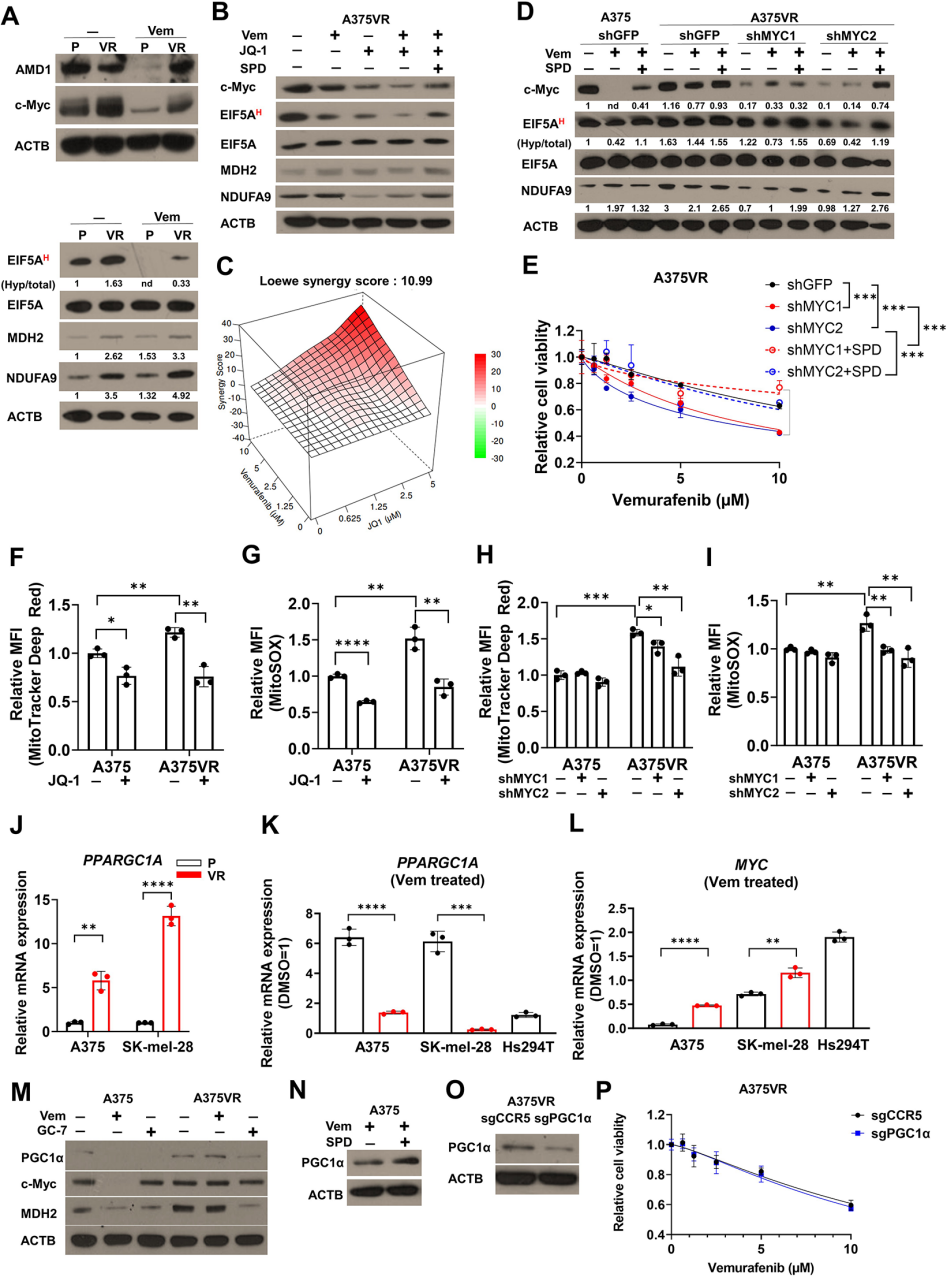
Persistent c-Myc activation underlies enhanced polyamine biosynthesis in vemurafenib resistant melanoma **A** Immunoblots of EIF5A hypusination, c-Myc, and mitochondrial proteins in A375 and A375VR cells treated with vemurafenib (1µM) for 48 h. **B** Immunoblots of A375VR cells treated with indicated drug combinations (Vem: 1µM, JQ-1: 1µM, SPD: 10µM) for 48 h. **C** Drug synergy score (using Loewe model) of vemurafenib and JQ-1 in A375VR. **D** Western blot analysis of A375VR cells treated with indicated shRNA and indicated materials (Vem: 1µM, SPD: 10µM) for 48 h. **E** Cell viability assay with A375VR expressing indicated shRNA treated with SPD (10µM) or PBS (*n* = 3) for 72 h. **F-G** Mitotracker deep red staining (F) and MitoSOX staining (G) with A375VR cells treated with JQ-1 (1µM) for 24 h (*n* = 3). **H-I** Mitotracker deep red staining (H) and MitoSOX staining (I) with A375VR cells expressing indicated shRNAs. shGFP was used as a negative control. **J** mRNA expressions of PPARGC1A (PGC1α) in parental and VR cells of A375 and SK-mel-28 (*n* = 3). **K-L** Fold change in PGC1α (**K**) and c-Myc (**L**) mRNA expression upon vemurafenib treatment (1µM) for 24 h (*n* = 3). **M** Immunoblots of A375VR cells treated with indicated drugs (Vem: 1 µM, GC-7: 10µM) for 48 h. **N** Immunoblots of A375 cells treated with indicated drugs for 48 h (Vem: 1µM, SPD: 10µM). **O** Immunoblots of A375VR-Cas9 cells expressing indicated sgRNA. **P** Cell viability assay after vemurafenib treatment for 72 h in A375VR-Cas9 cells expressing indicated sgRNAs. Numbers below blots in **A** and **D** indicate normalized densitometry values calculated by Image J. All plots indicate mean ± s.d. Student’s t-test was used to determine statistical significance for **E-L**. *, *p* < 0.05; **, *p* < 0.01; ***,*p* < 0.001; ****,*p* < 0.0001

We investigated the mechanism underlying upregulation of polyamine biosynthesis in vemurafenib-resistant melanoma by first examining whether established vemurafenib resistance signaling, such as ERK or AKT, is responsible for increased EIF5A hypusination. Notably, inhibition of ERK or AKT led to decreased EIF5A hypusination in both A375VR and SK-mel-28VR (Figs. S5A-B). Conversely, inhibition of EIF5A hypusination by GC-7 or DFMO did not affect ERK or AKT activity (Figs. S5C-D). These results place polyamine biosynthesis and EIF5A hypusination downstream of ERK and AKT signaling in vemurafenib resistance. Previous study identified c-Myc as critical shared downstream factor that integrates multiple vemurafenib resistance mechanisms including ERK, AKT and NOTCH signaling [49]. Given that c-Myc upregulates polyamine biosynthesis [50, 51], our results suggested that polyamine biosynthesis and EIF5A hypusination as critical targets of c-Myc driven vemurafenib resistance. Notably, while c-Myc, EIF5A hypusination were greatly reduced in A375 and SK-mel-28 parental cells upon vemurafenib treatment, this decrease was attenuated in A375VR and SK-mel-28VR (Fig. 4A, S5E). In line with prior studies demonstrating that c-Myc promotes *ODC1* transcription [51], the mRNA expression of *ODC1* and other polyamine biosynthesis genes, including *AMD1, SRM, SMS* and *DHPS*, decreased with treatment with JQ-1, a BET bromodomain inhibitor against c-Myc-dependent transcription coactivation [52] (Fig. S5F). JQ-1 also reduced EIF5A hypusination and the expression of mitochondrial proteins (Fig. 4B). The combination of JQ-1 and vemurafenib synergistically killed A375VR, Hs294T and SK-mel-28VR cells (Fig. 4C, S5G). Similarly, c-Myc knockdown with short hairpin RNA (shRNA) in A375VR cells decreased EIF5A hypusination and mitochondrial proteins (Fig. 4D) and sensitized them to vemurafenib (Fig. 4E). Spermidine supplementation rescued the effect of JQ-1 (Fig. 4B) or c-Myc knockdown (Fig. 4D-E), suggesting that altered polyamine biosynthesis plays a major role in c-Myc-mediated vemurafenib resistance. We also confirmed that the vemurafenib + trametinib combination failed to mitigate c-Myc-dependent EIF5A hypusination and upregulation of mitochondrial proteins in A375VR and SK-mel-28VR cells (Figs. S5H-I), suggesting that the c-Myc/polyamine-driven drug resistance program can be an attractive target in combination with BRAF and MEK inhibitors, as we have shown above (Fig. 1G). Consistent with Fig. 3K-L, mitochondrial content and mitochondrial superoxide were reduced by JQ-1 (Fig. 4F-G) or c-Myc knockdown (Fig. 4H-I) in A375VR cells.

BRAF inhibitor dabrafenib and trametinib combination is popularly used in clinic and showed superior efficacy over BRAFi monotherapy [53]. We extended our investigation of mechanisms governing drug resistance by examining the contribution of polyamine biosynthesis and EIF5A hypusination to resistance to dabrafenib + trametinib combination. Expectedly, A375VR and SK-mel-28VR cells are resistant to dabrafenib monotherapy and dabrafenib + trametinib combination (Figs. S6A-D). Pharmacological inhibition of AMD1, ODC1, c-Myc and DHPS sensitized vemurafenib resistant cells to dabrafenib and dabrafenib + trametinib combination (Figs S6A-D). Consistent with figure S5H-I, A375VR and SK-mel-28VR cells had sustained c-Myc protein levels and EIF5A hypusination upon dabrafenib and dabrafenib + trametinib treatment (Figs. S6E-F). Collectively, our results demonstrate that the regulation of polyamine biosynthesis and EIF5A hypusination downstream of c-Myc is responsible for resistance to not only BRAFi but also BRAFi + MAPKi combination used in clinic.

PGC1α is known as a master regulator of mitochondrial biogenesis and is associated with drug resistance in melanoma [21, 24]. Additionally, recent evidence suggests mutual antagonism between c-Myc and PGC1α in regulating the metabolic phenotype and drug response [54]. Therefore, we explored the interactions between c-Myc and PGC1α in vemurafenib resistance in melanoma. Basal PGC1α mRNA expression levels were higher in vemurafenib-resistant A375VR and SK-mel-28VR cells than in their respective parental cells (Fig. 4J). However, the induction of PGC1α mRNA expression [24, 55] upon vemurafenib treatment was observed only in vemurafenib-sensitive cell lines, and such increase was completely abrogated in their vemurafenib resistant counterparts (Fig. 4K). In line with its mutual antagonism with PGC1α, c-Myc expression was much more efficiently sustained in vemurafenib resistant cells compared to those in their respective parental cells (Fig. 4L). Intriguingly, PGC1a protein level was markedly decreased with vemurafenib treatment in vemurafenib-sensitive cells, while this level was sustained in vemurafenib-resistant A375VR and Hs294T cells (Fig. 4M, S7A). This discrepancy between PGC1α mRNA and protein levels suggested that there may be a posttranscriptional regulation of PGC1α protein levels. Consistent with this, the mRNA expressions of downstream targets of PGC1α, including *NRF1* and *IDH3A* were decreased with vemurafenib treatment only in A375 parental cell but not in A375VR (Figs. S7B, C). Notably, GC-7 treatment decreased PGC1α protein levels, suggesting that it may be modulated by EIF5A hypusination (Fig. 4M). Other polyamine biosynthesis inhibitors, such as DFMO or sardomozide, also similarly decreased PGC1α protein levels (Fig. S7D). In addition, spermidine restored PGC1α protein levels reduced by vemurafenib in A375 and SK-mel-28 cells (Fig. 4N, S7E). These findings suggest that a posttranscriptional regulation of PGC1α is responsible for its protein abundance in vemurafenib resistant cells, and that this regulation is dependent on polyamine biosynthesis and c-Myc (see discussion below). Nonetheless, PGC1α knockout failed to reverse resistance to vemurafenib in A375VR cells (Fig. 4O-P). These data suggest that, at least in the context of BRAF mutated melanoma, c-Myc plays a dominant role in conferring vemurafenib resistance through the EIF5A hypusination-mitochondrial activity axis.

### Targeting polyamine biosynthesis synergizes with BRAF inhibitors *in vivo*

**Fig. 5:**
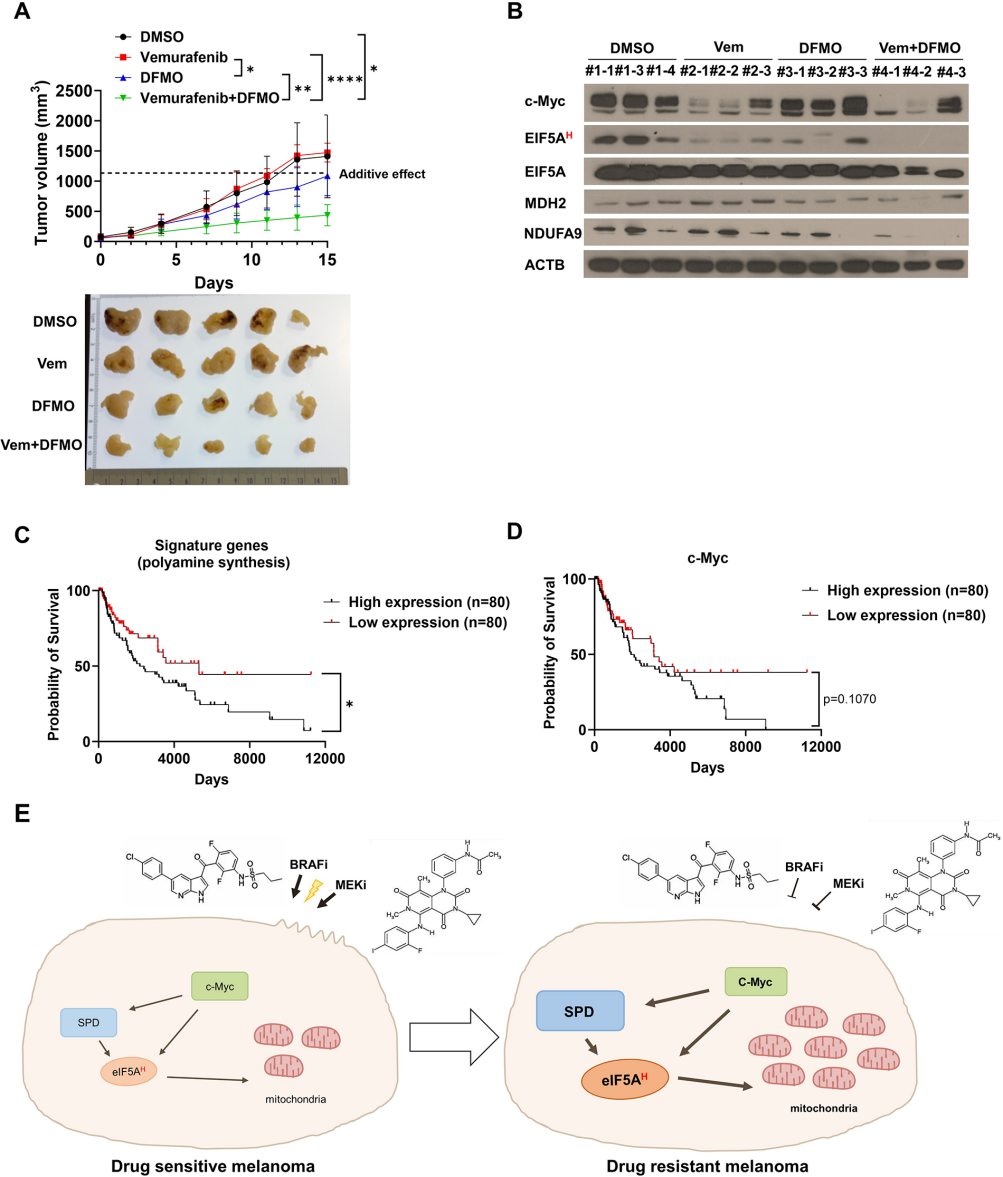
Inhibition of polyamine biosynthesis synergizes with vemurafenib in treating BRAF mutant melanoma*in vivo*. **A** Tumor volume of A375VR xenograft model (*n* = 5). Vemurafenib: 20 mg/kg, DFMO: (2% w/v) in drinking water. Additive effect was calculated with Bliss independence model. Student’s t-test was used to determine statistical significance. **B** Western blot analysis in xenograft tumors treated with indicated drug combinations for 15 days. **C** Kaplan-Meier curves for BRAF V600E melanoma patients in TCGA classified with polyamine synthesis signature score. **D** Kaplan-Meier curves for patients in (C) classified with c-Myc expression level. **E** Schematic diagram of c-Myc-polyamine axis promoting vemurafenib resistance. All plots indicate mean ± s.d. *, *p* < 0.05; **, *p* < 0.01; ***,*p* < 0.001; ****,*p* < 0.0001

To validate our observations in vivo, we established an A375VR melanoma xenograft mouse model and treated it with vemurafenib and DFMO. Consistent with our previous findings, the combination of vemurafenib and DFMO demonstrated a synergistic effect in vivo, resulting in a significant delay in tumor growth relative to the control group treated with either drug alone (Fig. 5A). The drug combination had no overt side effects, with no significant weight loss in any of the treatment groups (Fig. S8A). Furthermore, EIF5A hypusination and the protein expression levels of MDH2 and NDUFA9 decreased in tumors treated with the combination of vemurafenib and DFMO, suggesting that the EIF5A hypusination-mitochondrial activity axis contributes to vemurafenib resistance in a xenograft model (Fig. 5B).

To further investigate the clinical relevance of our findings, we analyzed the RNA sequencing results of BRAF V600E mutant skin cutaneous melanoma (SKCM) in The Cancer Genome Atlas (TCGA) database [56]. Integration of GTEx (Genome-Tissue Expression) and TCGA data in OncoDB [57] revealed that the expression of AMD1 was elevated in SKCM compared to normal tissue (Fig. S8B). Notably, the enrichment of the polyamine synthesis signature consisting of the mRNA expression levels of key polyamine synthesis-related genes, including ODC1, AMD1, SRM, SMS, DHPS, DOHH, and PAOX, was correlated with poor prognosis (Fig. 5C). The expression of c-Myc also had a weak correlation with poor prognosis (*P* = 0.1070) (Fig. 5D), while that of PGC1α had no relation with patient prognosis (Fig. S8C). Additionally, by analyzing RNA sequencing data in the CCLE (Cancer Cell Line Encyclopedia) [58] database, we confirmed that c-Myc target and OXPHOS-related genes are highly upregulated in vemurafenib-resistant cell lines (Fig. S8D). Collectively, these findings provide compelling evidence that blocking polyamine synthesis can effectively overcome resistance to vemurafenib by modulating the EIF5A hypusination-mitochondrial respiration pathway in vivo.

## Discussion

In this study, we identified a novel mechanism involving c-Myc, polyamine biosynthesis, EIF5A hypusination and mitochondrial respiration as critical mediators of vemurafenib resistance. Vemurafenib-resistant cancers exhibit a higher polyamine biosynthesis rate and subsequent EIF5A hypusination and translation of mitochondrial proteins. Key molecular targets in this process, including ODC1, AMD1, DHPS and c-Myc could serve as effective drug targets addressing specific vulnerabilities in BRAFi resistant melanoma, warranting development of novel breakthrough therapies against therapy resistant melanoma.

Our works suggest an intriguing possibility that polyamine metabolism may also be part of metabolic rewiring during the emergence of BRAFi and MAPKi resistant melanoma. Indeed, BRAFi and MAPKi treated melanoma enters ‘starved-like’ state with increased OXPHOS and reduction in glucose uptake and glycolysis [59]. Especially notable is the dramatic increase in intracellular polyamine levels observed upon vemurafenib treatment in vemurafenib resistant cancer cells (Fig. 2B). While c-Myc, MAPK and AKT signaling may be responsible for increased polyamine biosynthesis, these pathways are not hyperactivated above baseline upon BRAFi and MAPKi treatment (Figs. S1E, S5H-I). This suggests that crosstalk with other signaling pathway or metabolic rewiring may regulate hyperaccumulation of polyamine upon BRAFi treatment. Consistently, polyamine transport is significantly increased in BRAFi resistant melanoma [60]. Amino acid metabolism including serine, glutamine, and methionine metabolism are associated with drug resistance [61]. Determining the interactions between polyamine biosynthesis and other metabolic alterations leading to BRAFi and MAPKi resistance will be essential for design of effective therapeutic strategies for minimizing drug resistance.

Our work identifies multiple therapeutic strategies that delay melanoma recurrence after BRAF and/or MAPK inhibitor therapy. While polyamines have been known as classic oncometabolites for several decades, their modulation for cancer therapy has largely been unsuccessful. For example, DFMO is listed on the World Health Organization’s List of Essential Medicines for the treatment of trypanosomiasis, but its use as a cancer therapy as a single agent was at best only modestly effective [62]. Our work shows that DFMO and other polyamine biosynthesis modulators synergize with BRAF inhibitors, suggesting that polyamine biosynthesis modulators can be an effective therapy when used selectively in BRAF/MAPK inhibitor-resistant tumors. The recent discovery of allosteric inhibitors of DHPS also suggests an intriguing possibility that they can also be used in combination with BRAF and MAPK inhibitors for synergistic efficacy [63, 64]. Modulation of c-Myc activity, which we and others identify as the key molecule inducing BRAFi resistance [49, 65], or OXPHOS can also be an attractive alternative in maximizing the therapeutic benefit of BRAF inhibitors.

We expect targeting polyamine biosynthesis and EIF5A hypusination is broadly effective against BRAFi resistant tumor of diverse underlying genetic cause. Two key works support our findings; (1) the enrichment of polyamine biosynthesis signatures in BRAFi/MAPKi resistant tumors regardless of the presence of key resistance gene mutations (Fig. 2I). Likewise, enrichment of c-Myc and mitochondrial OXPHOS related genes are also found in BRAF mutant melanoma with intrinsic resistance (Fig. S8D). (2) The decrease in c-Myc and EIF5A hypusination upon ERK or AKT inhibitor treatment (Fig, S5A-B). As c-Myc has been demonstrated as a convergent downstream of diverse vemurafenib resistance pathways [49], we believe polyamine biosynthesis and EIF5A hypusination presents generalizable druggable targets that slow recurrence of BRAFi therapy resistant melanoma with diverse genotypes.

Inhibition of polyamine biosynthesis may provide effective synergistic therapy generalizable for diverse tumor types other than melanoma with BRAF mutations. In addition to melanoma, colorectal cancer and thyroid cancer are also known to frequently carry BRAF mutations [66]. However, BRAF inhibitors have proven largely ineffective against those tumors [67]. Notably, c-Myc is amplified in colon cancer, and polyamine and c-Myc are known to promote colorectal cancer cell survival [68], suggesting that targeting polyamine and c-Myc may contribute to BRAFi resistance in colon cancer. Indeed, recent reports suggest that inhibition of EIF5A hypusination with GC-7 can inhibit colorectal cancer growth [19]. These findings suggest that blocking the polyamine synthesis pathway would be a therapeutic strategy for c-Myc-amplified or BRAF-mutant cancer.

Our work identified the c-Myc-polyamine biosynthesis axis as a modulator of mitochondrial activity. This was surprising considering the central role of PGC1α in modulating mitochondrial biogenesis and metabolic plasticity. Of note, previous studies reported upregulation of PGC1α mRNA regardless of vemurafenib resistance. Therefore, it is likely that PGC1α mRNA accumulation is a primary response to BRAF inhibitors and may not be causal or sufficient for establishing drug resistance. Instead, we show evidence of posttranscriptional upregulation of PGC1α protein by polyamine in vemurafenib-resistant melanoma. Inhibition of polyamine biosynthesis or EIF5A hypusination decreased PGC1α protein levels while not influencing PGC1α mRNA levels (Fig. 4M, S7A). The exact molecular mechanisms underlying the regulation of PGC1α protein levels are currently unclear. PGC1α is known to be posttranscriptionally regulated by many signaling pathways, including the PI3K/AKT and p38 signaling pathways [69]. Polyamines and EIF5A hypusination may regulate these key posttranscriptional regulators of PGC1α. Elucidation of the exact molecular mechanisms of polyamine mediated PGC1α regulation and discovery of possible druggable interventions in this process warrant further study.

Further works should assess the contribution of our newly identified mechanisms involving polyamine biosynthesis and EIF5A hypusination in the context of dynamic rewiring and adaptation of BRAF mutant melanoma upon BRAFi/MAPKi treatment. Accumulating evidence suggest that BRAFi and MAPKi treated melanoma cells undergo extensive adaptive transcriptional response to enter drug tolerant state. This state involves many non-mutational changes with heterogenous outcome, such as upregulation of MITF, activation of SOX10 and NGFR positive neural crest stem cell state, and AXL positive invasive property [70–72]. Metabolic plasticity of melanoma cells upon BRAFi and MAPKi treatment including increased mitochondrial biogenesis, increased fatty acid oxidation is also critical for drug tolerance [26]. As our CRISPR screening and delineation of the downstream mechanisms underlying BRAFi and MAPKi resistance was limited to the cell line models that already established acquired resistance (e.g. A375VR, SK-mel-28VR) or intrinsic resistance (Hs294T) to therapy, future studies should elucidate metabolic rewiring involving polyamine biosynthesis, EIF5A hypusination and increased mitochondrial biogenesis in the complex process of adaptation, plasticity and acquisition of resistance in BRAFi and MAPKi treated melanoma.

## Conclusions

Our work delineates a novel mechanism underlying BRAFi resistance in melanoma. Vemurafenib resistant melanoma has markedly upregulated polyamine biosynthesis rate, which in turn induces EIF5A hypusination, translation of mitochondrial proteins and enhanced mitochondrial activity. c-Myc activation is primarily responsible for increased polyamine biosynthesis in vemurafenib resistant melanoma. Pharmacological and genetic disruption of key polyamine biosynthesis enzymes including AMD1 and ODC1, and c-Myc and mitochondrial oxidative phosphorylation selectively kills multiple cell line models of intrinsic and acquired vemurafenib resistance. Our result identifies druggable therapeutic targets that can be targeted to overcome genetically heterogeneous BRAFi resistance.

### Electronic supplementary material

Below is the link to the electronic supplementary material.

Supplementary Material 1

Supplementary Material 2

## Data Availability

The NGS data is available in NCBI GEO with accession number GSE243344. The custom code used to analyze NGS data is available in Github (https://github.com/tackhoonkim).

## Abbreviations

DFMO: DL-α-difluoromethylornithine
MITF: Microphthalmia-associated transcription factor
PGC1α: Peroxisome proliferator-activated receptor gamma coactivator 1-alpha
ROS: Reactive oxygen species
AMD1: S-adenosylmethionine decarboxylase 1
CRISPR: Clustered regularly interspaced short palindromic repeats
sgRNA: Single guide RNA
DHPS: Deoxyhypusine synthase
MTS: Mitochondrial targeting sequence
shRNA: Short hairpin RNA
TCGA: The cancer genome atlas
SKCM: Skin cutaneous melanoma
OXPHOS: Oxidative phosphorylation
NGS: Next Generation Sequencing
NCBI: National Center for Biotechnology Information
GEO: Gene Expression Omnibus
