# Supplementary material for: Polyamine and EIF5A hypusination downstream of c-Myc confers targeted therapy resistance in BRAF mutant melanoma
Source: Mol Cancer. 2024 Jul 4;23:136. doi: 10.1186/s12943-024-02031-w (PMC11223307; doi:10.1186/s12943-024-02031-w)
Supplement: Supplementary file 1 — Supplementary Material 1 [file 12943_2024_2031_MOESM1_ESM.docx]

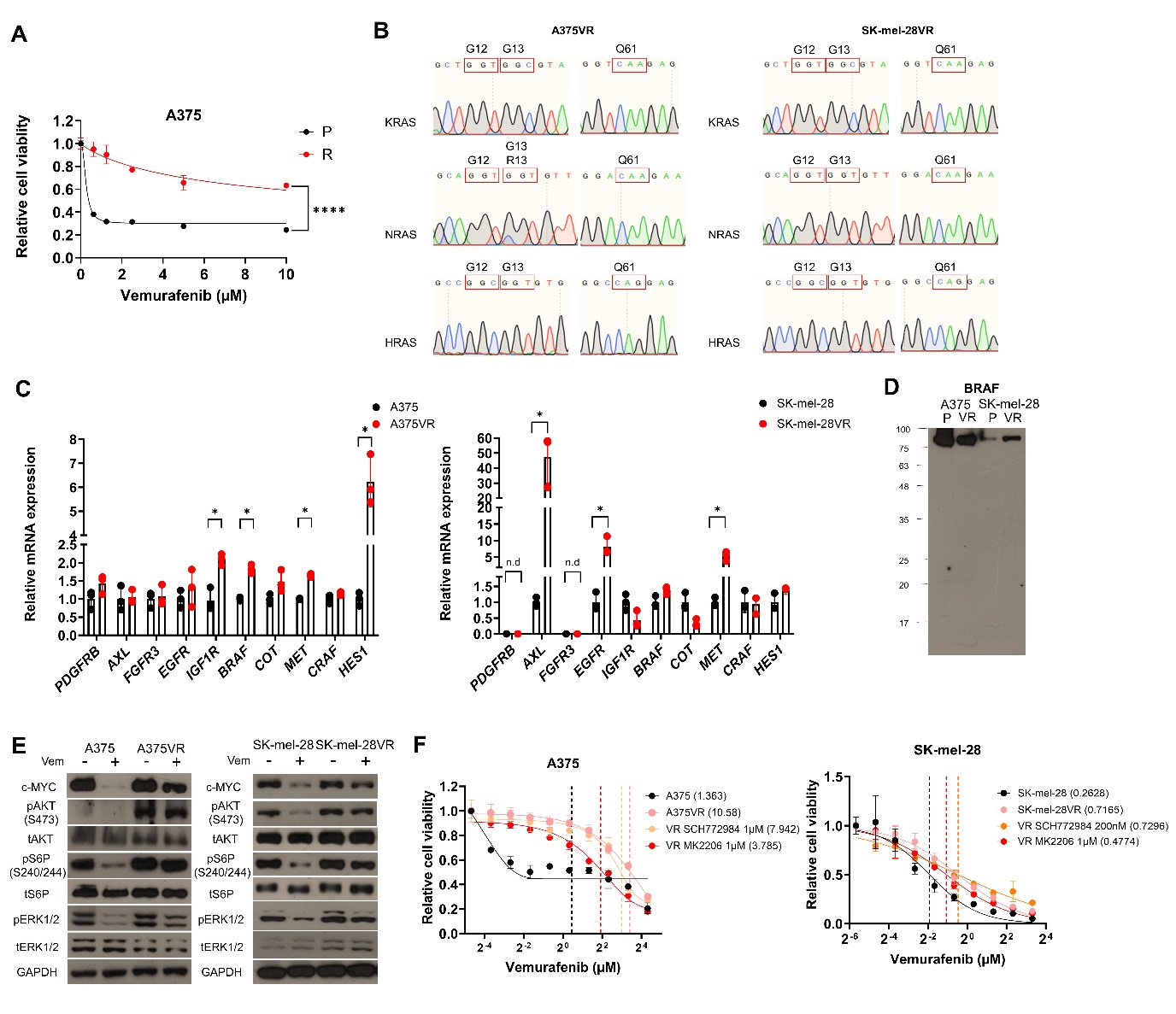


**Figure S1.** **Characterization of BRAF V600E mutant cell lines with acquired vemurafenib resistance.** **A** Cell viability assay of A375 and A375VR cells treated with vemurafenib for 72hrs at indicated concentrations (n=3). **B** Sanger sequencing results of select mutational hotspots of RAS mRNAs in A375VR and SK-mel-28VR. **C** RT-qPCR analyses of indicated genes in A375VR and SK-mel-28VR**.** n.d, not detected. **D** Immunoblot data of A375 and SK-mel-28 for detection of N-terminal truncated BRAF splice variants. **E** Immunoblot data of A375 and SK-mel-28 after vemurafenib treatment for 48hrs. **F** Cell viability assay of A375 and A375VR (left) and SK-mel-28 and SK-mel-28VR (right) treated with vemurafenib in the presence and absence of SCH772984 or MK2206 for 72hrs. Dashed vertical lines indicate calculated vemurafenib GI50 concentration, which is also noted in legend in parentheses in μM.. Student’s t-test was used for calculating significance in **C**. All plots indicate mean ± s.d. *, p<0.05; **, p<0.01; ***,p<0.001; ****,p<0.0001.


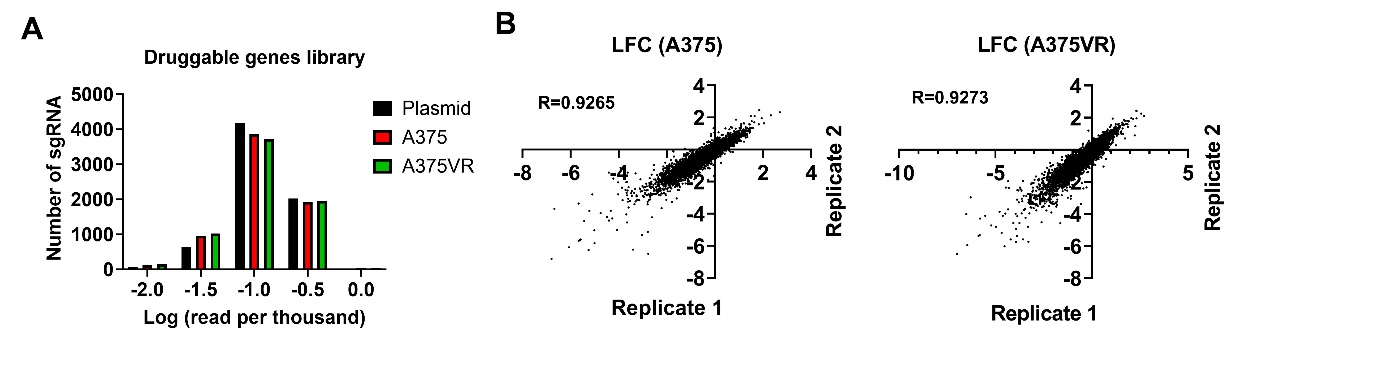


**Figure S2. Data quality control of CRISPR-cas9 screens in figure 1. A** Frequency distribution of sgRNA in the sgRNA library in indicated samples. **B** Correlation of log fold change (LFC) of sgRNA frequency between two biological replicates.

**
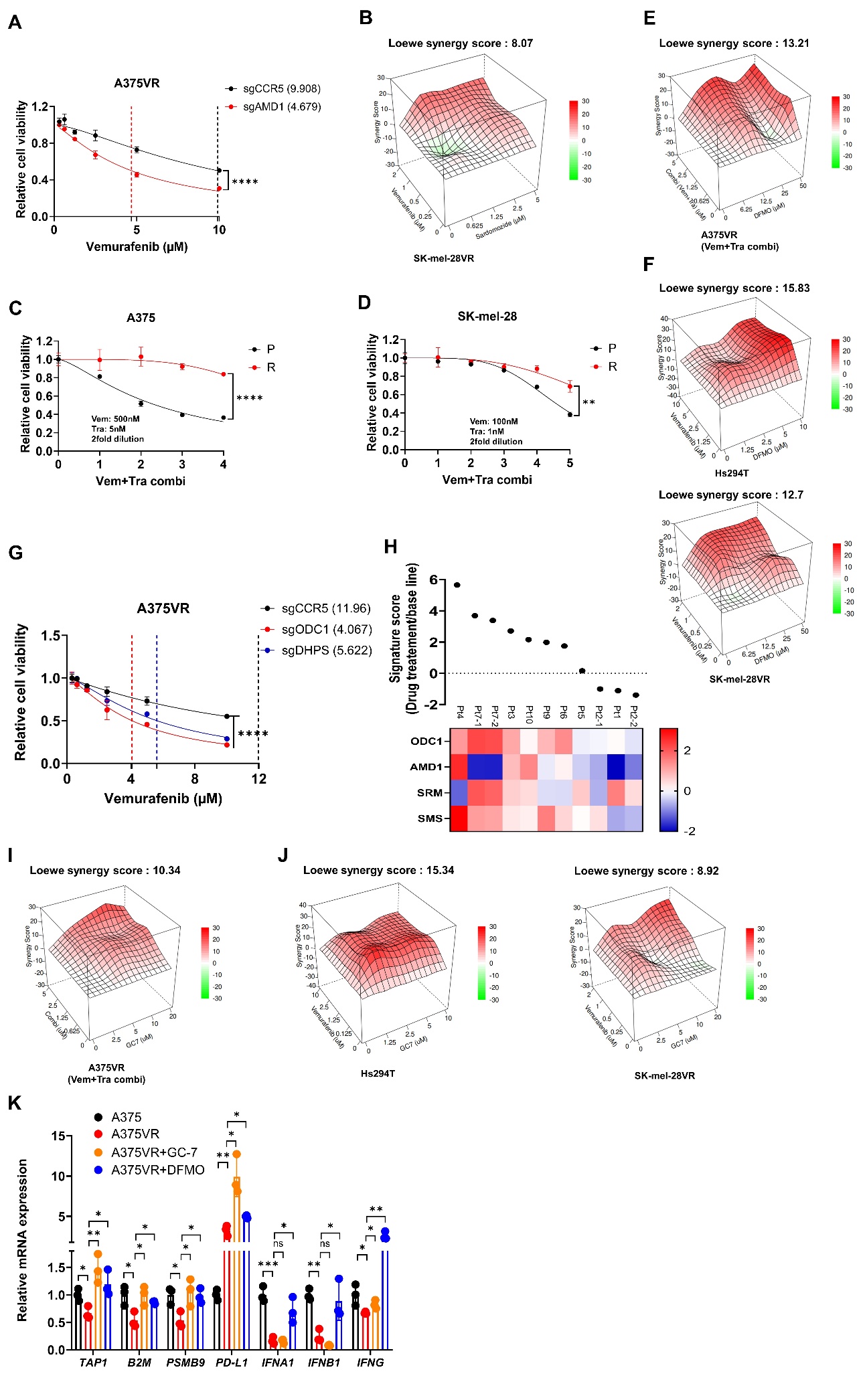
**

**Figure S3. Synergistic drug interaction of BRAF/MEK inhibitor and polyamine biosynthesis and EIF5A hypusination inhibitors.** **A** Dose-response assay for vemurafenib of A375VR Cas9 cells expressing indicated sgRNA (n=3). Dashed vertical lines indicate calculated vemurafenib GI50 concentration, which is also noted in legend in parentheses in μM. **B** Drug synergy score calculated by synergyfinder in SK-mel-28VR. **C-D** Combinatorial drug treatment of vemurafenib and trametinib in parental (P) or vemurafenib resistant (R) A375 (C) and SK-mel-28 (D). Vemurafenib and trametinib were treated at concentrations of 2-fold dilution from 500nM (Vem) and 5nM (Tra) (C) or 100nM (Vem) and 1nM (Tra) (D) (n=3).  **E-F** Synergyfinder analysis with BRAF/MEK inhibitor in combination with DFMO in indicated vemurafenib resistant cell lines. “Combi” indicates the combination of vemurafenib and trametinib treated in 2-fold serial dilution starting from 5µM (vemurafenib) and 5nM (trametinib) . **G** Dose-response assay for vemurafenib of A375VR Cas9 cells expressing indicated sgRNA (n=3). Dashed vertical lines indicate calculated vemurafenib GI50 concentration, which is also noted in legend in parentheses in μM. **H** Sum of log fold changes (LFC) of polyamine synthesis related genes after dabrafenib and trametinib in the previous published patient data (GSE61992). **I-J** Synergyfinder analysis with BRAF/MEK inhibitor in combination with GC-7. “Combi” indicates the combination of vemurafenib and trametinib treated in 2-fold serial dilution starting from 5µM (vemurafenib) and 5nM (trametinib). **K** Relative mRNA expression levels of indicated genes for the treatment of GC-7 (10µM) and DFMO (200µM). All plots indicate mean ± s.d. All drugs were treated for 72hrs (**A-G, I-J**) and 24hrs (**K**). Student’s t-test was used to determine statistical significance for **A, C-D, G, K**. All plots indicate mean ± s.d. *, p<0.05; **, p<0.01; ***,p<0.001; ****,p<0.0001.


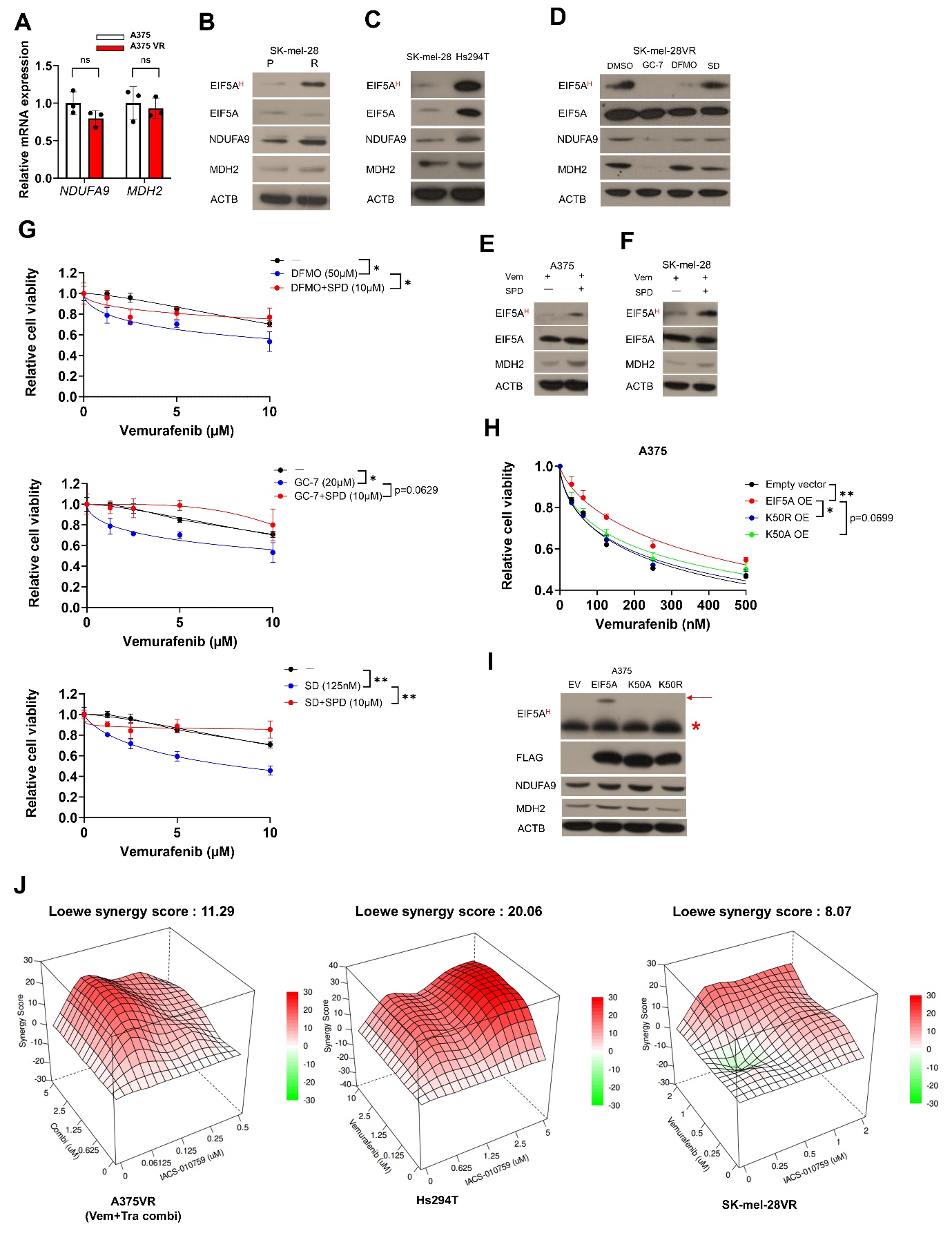


**Figure S4. EIF5A hypusination and subsequent enhancement of mitochondrial activity promotes vemurafenib resistance.** **A** Relative mRNA expression levels of *MDH2* and *NDUFA9* in A375 and A375VR (n=3). **B-C** Immunoblotting of EIF5A hypusination (EIF5A^H^) and mitochondrial proteins in indicated cell lines. **D** Immunoblotting of EIF5A hypusination (EIF5A^H^) and mitochondrial proteins in SK-mel-28VR with treatment of indicated drugs for 48hrs. GC-7: 10µM, DFMO: 10µM, SD: 2µM. **E-F** Immunoblotting of A375 and SK-mel-28 treated with vemurafenib (Vem 1µM in e, 100nM in f) and spermidine (SPD, 10µM) for 48hrs. **G** Cell viability assay with A375VR cells treated with indicated drug combinations and spermidine supplementation for 72hrs (n=3). **H-I** A375 cell lines that overexpress EIF5A (WT), EIF5A (K50A) or EIF5A (K50R) were generated. **H** Cell viability assay of indicated cells treated with vemurafenib for 72hrs (n=3). **I** Immunoblotting with the cells assayed in (H). Red arrow indicates exogenous hypusinated EIF5A and red asterisk indicates endogenous hypusinated EIF5A. **J** Drug synergy score of IACS-010759 and vemurafenib or vem+tra combination in indicated cell lines. All drugs were treated for 72hrs (**J**). Student’s t-test was used for calculating significance (**A, G** and **H**). All plots indicate mean ± s.d. *, p<0.05; **, p<0.01; ***,p<0.001; ****,p<0.0001.

**
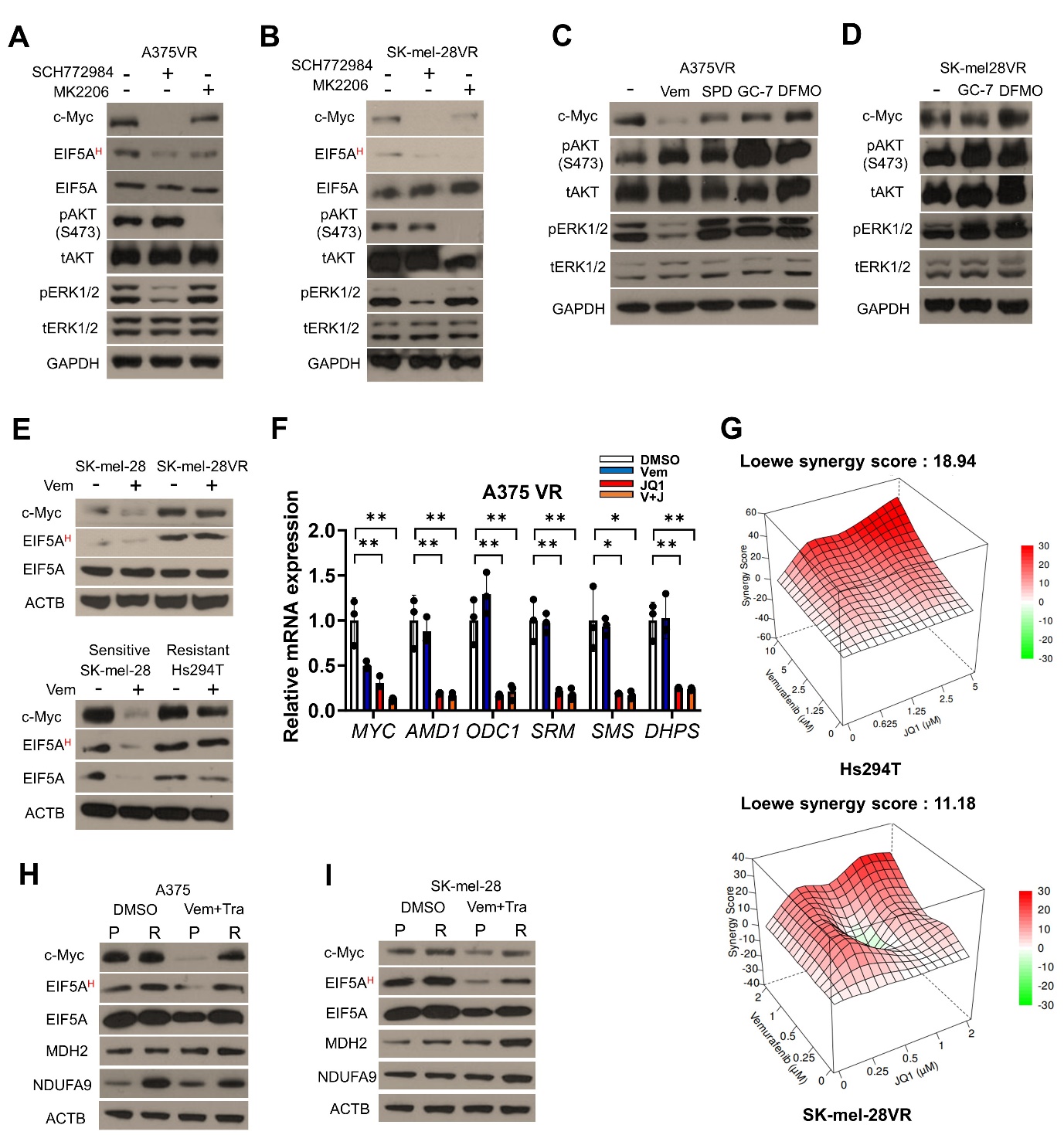
**

**Figure S5. Persistent c-Myc-EIF5A hypusination in vemurafenib resistant melanoma.** **A-D** immunoblots of A375VR and SK-mel-28VR treated with indicated drugs. **A-B** A375VR (A) and SK-mel-28VR (B) were treated with SCH772984 (1μM) or MK2206 (1μM) for 4 hours. **C** A375VR cells were treated with vemurafenib (VEM, 1μM), spermidine (SPD, 10μM), GC-7 (10μM), or DFMO (200μM) for 48 hours. **D** SK-mel-28VR cells were treated with GC-7 (10μM) or DFMO (10μM) for 48 hours. **E** Immunoblots of c-Myc and hypusinated EIF5A (EIF5A^H^) in SK-mel-28, SK-mel-28VR and Hs294T treated with vemurafenib (100nM) for 48hrs. **F** Relative mRNA expressions of c-Myc and polyamine synthesis-related genes in A375VR with treatment of indicated drugs for 24hrs (n=3). **G** Drug synergy scores of vemurafenib and JQ-1 combination in indicated cell lines (vemurafenib and JQ-1 were treated for 72hrs). **H** Immunoblotting of A375 after combinatorial treatment of vemurafenib (1µM) and trametinib (1nM). **I** Immunoblots of SK-mel-28 after combinatorial treatment of vemurafenib (100nM) and trametinib (1nM). Vemurafenib and trametinib were treated for 48hrs (**H-I**). Student’s t-test was used for calculating significance (**F**). All plots indicate mean ± s.d. *, p<0.05; **, p<0.01; ***,p<0.001; ****,p<0.0001.


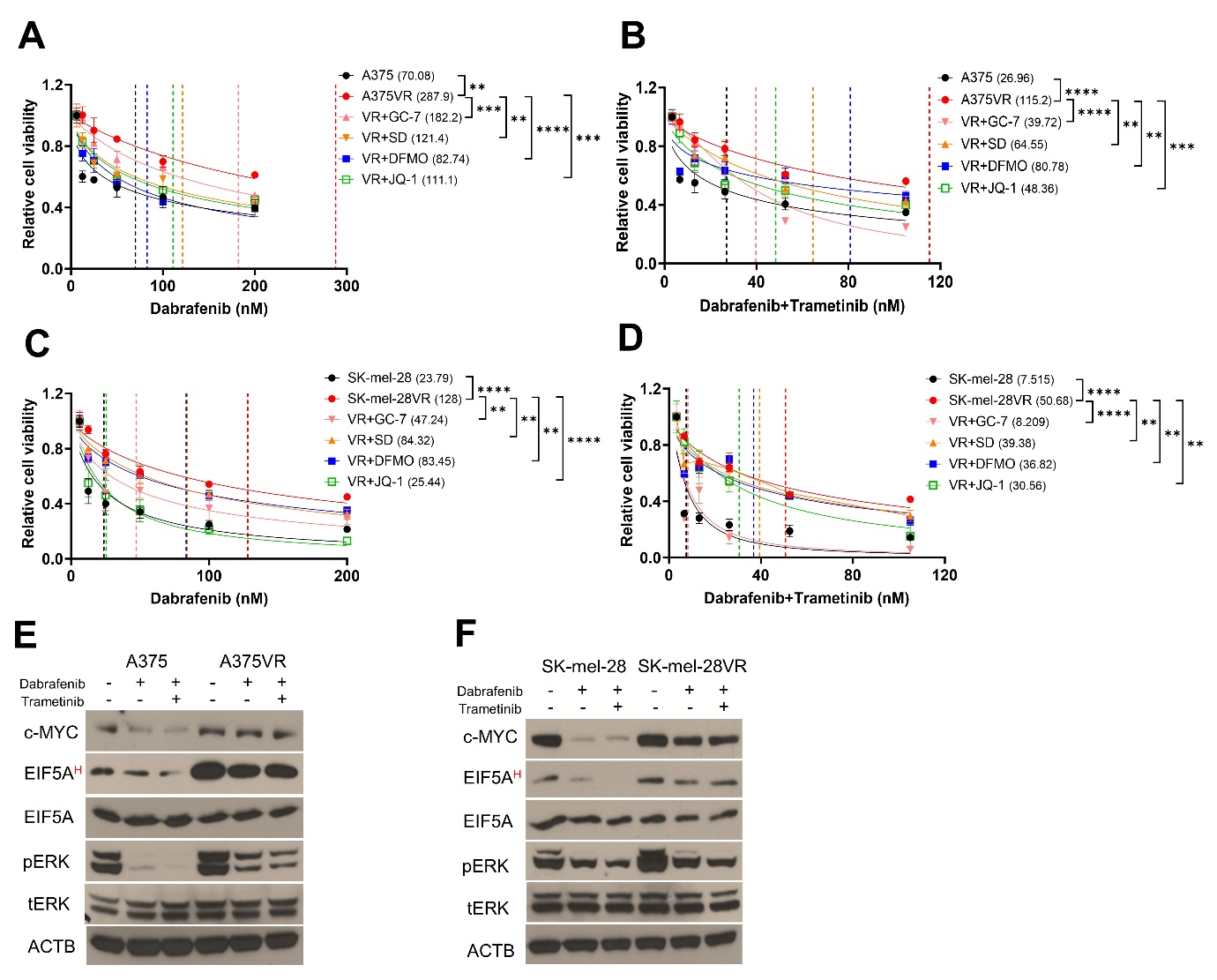


**Figure S6. Modulation of polyamine biosynthesis, EIF5A hypusination and c-Myc synergizes with dabrafenib and trametinib combinations in A375VR and SK-mel-28VR.** **A-D** Cell viability assay in A375 (A-B) and SK-mel-28 (C-D) treated with drug combinations of indicated drugs and dabrafenib or dabrafenib+trametinib combination (n=3). Dabrafenib+trametinib were treated at concentrations of 2-fold dilution from 100nM and 5nM (B, D) Dashed vertical lines indicate calculated GI50 concentration, which is also noted in legend in parentheses in nM. Student’s t-test was used for calculating significance. **E-F** Immunoblot analysis of c-Myc and EIF5A^H^ in A375 (E) and SK-mel-28 (F) (Dabrafenib: 100nM, trametinib: 1nM). All drugs were treated for 72hrs (**A-D**) and 48hrs (**E-F**). All plots indicate mean ± s.d. *, p<0.05; **, p<0.01; ***,p<0.001; ****,p<0.0001.


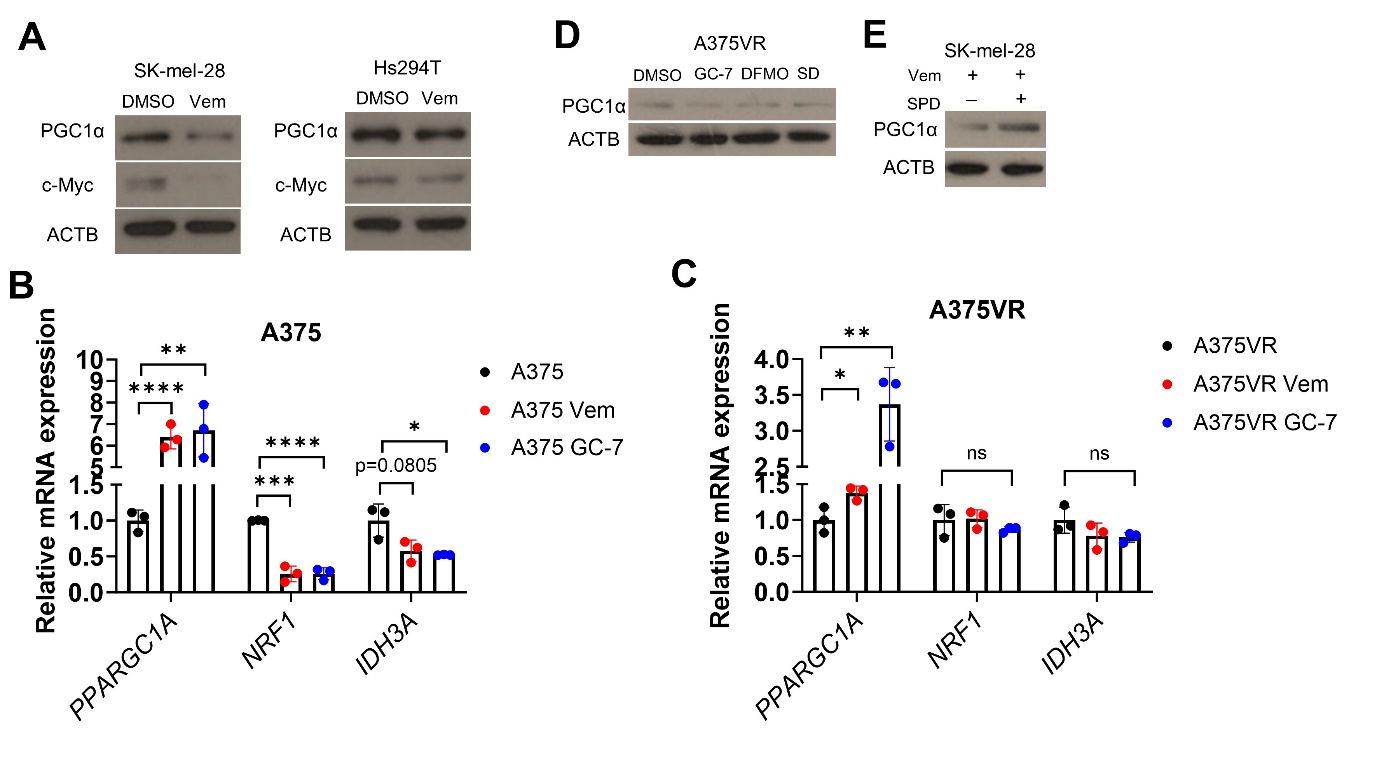


**Figure S7. PGC1α activity is sustained in A375VR.** **A** immunoblots of two cell lines with treatment of vemurafenib for. **B-C** Relative mRNA expressions of *PPARGC1A* (PGC1α) target genes in A375 and A375VR treated with vemurafenib (1µM) or GC-7 (10µM) for 24hrs (n=3). **D** immunoblots of A375VR treated with GC-7 (10µM), DMFO (100µM), or sardomozide (1µM). **E** immunoblots of SK-mel-28 treated with indicated materials (Vem: 100nM, SPD: 10µM). All drugs were treated for 48hrs (**A, D, E**) and 24hrs (**B, C**). Student’s t-test was used for calculating significance (**B-C**). All plots indicate mean ± s.d. *, p<0.05; **, p<0.01; ***,p<0.001; ****,p<0.0001. ns, p>0.05


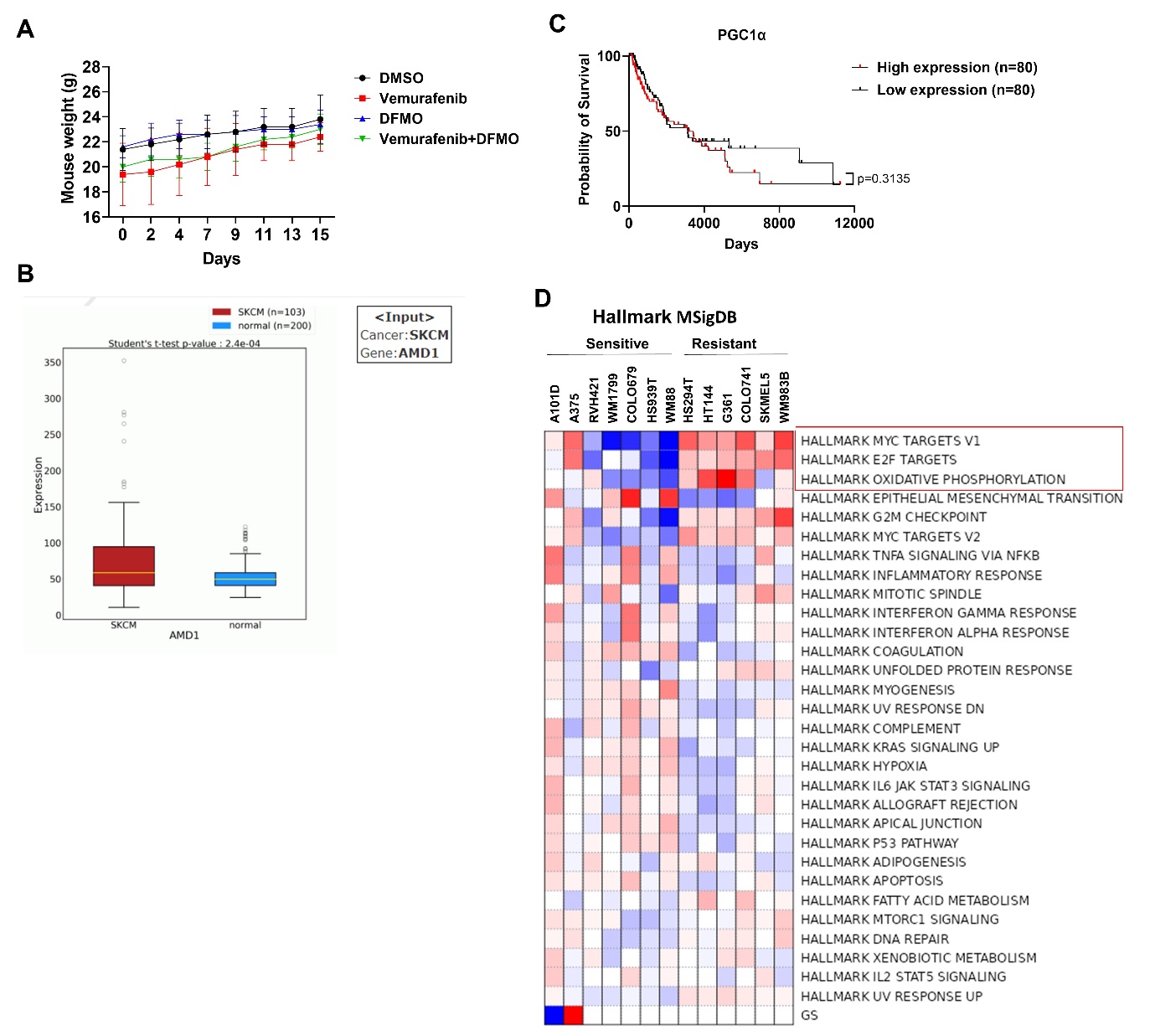


**Figure S8. Significance of c-Myc-polyamine biosynthesis in BRAF inhibitor resistance.** **A** Body weight of mice analyzed in figure 5A. **B** Box and whisker plots of expression levels of AMD1 in normal tissue and melanoma (Skin cutaneous melanoma, SKCM). **C** Kaplan-Meier curves for patients in figures 5C-D classified with PGC1α expression level. **D** Pathway analysis of BRAF V600E mutant melanoma cell lines in CCLE grouped by vemurafenib resistance.

**Table S1. sgRNAs and shRNAs used in the study**

| **sgRNA sequences (5'-3')** | **Description** |  |
| --- | --- | --- |
| AAGCTTGCTAGGGATTACAG | sgAMD1-1 | AMD1 knockout 1 |
| GGAATTCGTGACCTGATACC | sgAMD1-2 | AMD1 knockout 2 |
| CGGGGCTACGACTTCAACCG | sgDHPS | DHPS knockout |
| CAACGCTGGGTTGATTACGC | sgODC1 | ODC1 knockout |
| GTTGTGACTGCGACTGTGTG | sgPPARGC1A | PPARGC1A knockout |
| GGGCGAGGAGCTGTTCACCG | sgGFP | Negative control for KO |
| GGTGACAAGTGTGATCACTT | sgCCR5 | Negative control for KO |
|  |  |  |
| **shRNA knockdown (5'-3')** | **Description** |  |
| CAGTTGAAACACAAACTTGAA | shMYC-1 F | MYC knockdown 1 |
| CCTGAGACAGATCAGCAACAA | shMYC-2 F | MYC knockdown 2 |
| GCAAGCTGACCCTGAAGTTCAT | shGFP F | Negative control for KD |

**Table S2. qPCR primers used in the study**

| **Sequences (5'-3')** | **Description** |
| --- | --- |
| AGTCGGGTAATCAGTCAGCCA | qAMD1 F |
| ACTCTCACGAGTGACATCCTTT | qAMD1 R |
| GTTTTGCGGATTGCCACTGAT | qODC1 F |
| GCTCTTTCGCCCGTTCCAA | qODC1 R |
| GTGGTGGCCTATGCCTACTG | qSRM F |
| CTCCTGGAAGTTCGTGCTCG | qSRM R |
| TACATGCGAAAAACGTGTGGC | qSMS F |
| CTTCAGTACCGGGATACAGTCT | qSMS R |
| TTCCTGGGATATACATCCAACCT | qDHPS F |
| AATACGTCCACCATGTTGTGC | qDHPS R |
| TCCCTCCACTCGGAAGGAC | qMYC F |
| CTGGTGCATTTTCGGTTGTTG | qMYC R |
| TCTGAGTCTGTATGGAGTGACAT | qPPARGC1A F |
| CCAAGTCGTTCACATCTAGTTCA | qPPARGC1A R |
| AGGAACACGGAGTGACCCAA | qNRF1 F |
| TATGCTCGGTGTAAGTAGCCA | qNRF1 R |
| AGCCGGTCACCCATCTATGAA | qIDH3A F |
| TAGAGACACATGGTCGGACAT | qIDH3A R |
| CCGACGAGTAGTACAACACAGC | qNDUFA9 F |
| GCTTCCTTGGACAGTTGAGCA | qNDUFA9 R |
| GCCATGATCTGCGTCATTGC | qMDH2 F |
| CCGAAGATTTTGTTGGGGTTGT | qMDH2 R |
| GCAGTCAACTCCTGGACCACTA | qTAP1 F |
| CAAGGTTCCCACTGCTTACAGC | qTAP1 R |
| CCACTGAAAAAGATGAGTATGCCT | qB2M F |
| CCAATCCAAATGCGGCATCTTCA | qB2M R |
| CGAGAGGACTTGTCTGCACATC | qPSMB9 F |
| CACCAATGGCAAAAGGCTGTCG | qPSMB9 R |
| TGCCGACTACAAGCGAATTACTG | qPD-L1 F |
| CTGCTTGTCCAGATGACTTCGG | qPD-L1 R |
| AGAAGGCTCCAGCCATCTCTGT | qIFNA1 F |
| TGCTGGTAGAGTTCGGTGCAGA | qIFNA1 R |
| GTCACTGTGCCTGGACCATAG | qIFNB1 F |
| GTTTCGGAGGTAACCTGTAAGTC | qIFNB1 R |
| TCGGTAACTGACTTGAATGTCCA | qIFNG F |
| TCGCTTCCCTGTTTTAGCTGC | qIFNG R |
